# Adapting C_4_ photosynthesis to atmospheric change and increasing productivity by elevating Rubisco content in sorghum and sugarcane

**DOI:** 10.1073/pnas.2419943122

**Published:** 2025-02-11

**Authors:** Coralie E. Salesse-Smith, Noga Adar, Baskaran Kannan, Thaibinhduong Nguyen, Wei Wei, Ming Guo, Zhengxiang Ge, Fredy Altpeter, Tom E. Clemente, Stephen P. Long

**Affiliations:** ^a^Carl R. Woese Institute for Genomic Biology, University of Illinois at Urbana-Champaign, Urbana, IL 61801; ^b^Center for Advanced Bioenergy and Bioproducts Innovation, University of Illinois at Urbana-Champaign, Urbana-Champaign, IL 61801; ^c^Agronomy Department, Plant Molecular and Cellular Biology Program, Genetics Institute, University of Florida, Institute of Food and Agricultural Science, Gainesville, FL 32603; ^d^Center for Advanced Bioenergy and Bioproducts Innovation, University of Florida, Gainesville, FL 32603; ^e^Department of Agronomy and Horticulture, University of Nebraska-Lincoln, Lincoln, NE 68583; ^f^Center for Advanced Bioenergy and Bioproducts Innovation, University of Nebraska-Lincoln, Lincoln, NE 68583; ^g^Departments of Plant Biology and of Crop Sciences, University of Illinois at Urbana-Champaign, Urbana, IL 61801

**Keywords:** atmospheric change, future-proofing agriculture, Rubisco, C_4_ photosynthesis, sorghum and sugarcane

## Abstract

Meta-analyses and theory show that with rising atmospheric [CO_2_], Rubisco has become the greatest limitation to light-saturated leaf CO_2_ assimilation rates (*A*_sat_) in C_4_ crops. So would transgenically increasing Rubisco increase *A*_sat_ and result in increased productivity in the field? Here, we successfully overexpressed the Rubisco small subunit (*RbcS*) with Rubisco accumulation factor 1 (*Raf1*) in both sorghum and sugarcane, resulting in significant increases in Rubisco content of 13 to 25% and up to 90% respectively. *A*_sat_ increased 12 to 15% and Rubisco enzyme activity ~40% in three independent transgenic events of both species. Sorghum plants also showed increased speeds of photosynthetic induction and decreased bundle sheath leakiness. These improvements translated into average increases of 15.5% in biomass in field-grown sorghum and a 37 to 81% increase in greenhouse-grown sugarcane. This suggests a potential opportunity to achieve substantial increases in productivity of this key economically important clade of C_4_ crops, future proofing their value under global atmospheric change.

## Significance

The world is projected to need a 60% increase in food supply by 2050 (UN), and this must be achieved under conditions of global change without expanding onto yet more land. C_4_ crops, while few in number, account for a large proportion of agricultural productivity. We reason that rising atmospheric [CO_2_] has very recently made Rubisco, the enzyme used for all carbon fixation in plants, the greatest limitation to light-saturated photosynthesis in C_4_ crops. We demonstrate that transgenically increasing Rubisco content in sorghum and sugarcane, increases their photosynthetic efficiency and productivity, including in a field trial of sorghum. This shows a means to sustainably increase the productivity of this key group of crops.

While very few of the 161 crop species listed by UN-FAO use C_4_ photosynthesis, their importance and potential in terms of global food, feed, and biomass production is exceptional (1). For total global biomass harvested, sugarcane (*Saccharum* hybrids) is number one and maize (*Zea mays* L.) number one for seed harvested (1, 2). Sorghum (*Sorghum bicolor*) is the fourth most important cereal in global production, only behind, maize, rice, and wheat, but it is the major cereal of the semiarid tropics (1). While grown for biomass, the C_4_ crops energycane and Miscanthus show exceptional productivity (3–6). These crops all belong to the monophyletic grass tribe Andropogonae.

The leaves of C_4_ plants contain an outer layer of mesophyll cells surrounding the bundle sheath cells. CO_2_ diffuses from the substomatal cavity into the mesophyll cytoplasm, where it combines with phosphoenolpyruvate (PEP) to form oxaloacetate (OAA), catalyzed by cytoplasmic PEP carboxylase (PEPC). OAA is reduced to malate in the mesophyll chloroplasts and then diffuses to the bundle sheath where it is decarboxylated by NADP^+^-dependent malic enzyme (NADP-ME) creating a high [CO_2_] around Ribulose-1:5 bisphosphate carboxylase/oxygenase (Rubisco). The decarboxylation product, pyruvate, diffuses back to the mesophyll where, catalyzed by Pyruvate Pi dikinase (PPDK), it is phosphorylated to PEP at the expense of 2 ATP, completing the photosynthetic C_4_ cycle (7). This photosynthetic C_4_ cycle is in effect a light energy–driven CO_2_ pump that elevates the [CO_2_] around Rubisco to a level that is sufficient to competitively inhibit oxygenation, preventing photorespiration and avoiding the significant losses of photosynthetic efficiency that this causes in C_3_ species (8). Its benefits are such that considerable effort is being placed into engineering the C_4_ pathway into C_3_ crops (9–11). Tests under open-air conditions using Free-Air Concentration Enrichment (FACE) technology, have shown that higher [CO_2_] increases the photosynthetic efficiency, and in turn productivity, of C_3_ crops, but not these C_4_ crops (12–16). Thus, rising atmospheric [CO_2_] could progressively erode the current production advantage of C_4_ plants (17). However, analysis of metabolic and physiological limitations indicates that C_4_ plants could be modified to also gain photosynthetic efficiency at today’s and future atmospheric [CO_2_] (18–20).

Light-saturated leaf CO_2_ assimilation rates (*A*_sat_) in C_4_ plants rise sharply with intercellular CO_2_ concentration (*C*_i_) to a plateau, where *A*_sat_ remains constant with further increase in *C*_i_ (7). In maize crops grown in the field at both ambient and elevated atmospheric CO_2_, this transition was shown to occur at a *C*_i_ of about 120 μmol mol^−1^_,_ above which *A*_sat_ is [CO_2_]-saturated (15). A meta-analysis of 842 measured *A*–*C*_i_ responses in 49 C_4_ species showed the point of CO_2_ saturation is consistent across a broad range of C_4_ species, and unaffected by growth at either subambient or supra-ambient [CO_2_] (19). Because of their lower stomatal conductance (*g*_s_) relative to *A*_sat_, the drawdown of [CO_2_] between the atmosphere (*C*_a_) and intercellular air space (*C*_i_) is greater in C_4_ leaves, resulting in a ratio (*C*_i_/*C*_a_) of about 0.4, compared to 0.7 in C_3_ species. These ratios in non-water-stressed plants are apparently unaffected by the [CO_2_] of the growth environment (12). At today’s *C*_a_ of 428 μmol mol^−1^ (21), *C*_i_ in photosynthesizing C_4_ leaves should be about 170 μmol mol^−1^. Therefore, at current and future atmospheric [CO_2_], C_4_ photosynthesis is [CO_2_] saturated. Historically this was not the case. The average *C*_a_ of the last 40,000 y in which our crop ancestors evolved was 220 μmol mol^−1^ and assuming a *C*_i_/*C*_a_, of 0.4 under these conditions average *C*_i_ would have been 88 μmol mol^−1^, well below the indicated saturation point of about 120 μmol mol^−1^ (12, 22). At this *C*_i_ photosynthesis would be operating on the initial slope of the response of *A* to *C*_i_. The mechanistic steady-state mathematical model of C_4_ photosynthesis shows that this initial slope is determined by the activity of PEPC and mesophyll conductance (*g*_m_); *g*_m_ being the ease with which CO_2_ can diffuse from the substomatal cavity into the mesophyll cytoplasm (7). The plateau, however, is limited by the activity of Rubisco, and potentially electron transport (7). Recent non-steady-state metabolic modeling and measurements show Rubisco activity also having significant control over the efficiency of photosynthesis in fluctuating light (23, 24). Consistent with these predictions, transgenic knock-down studies showed metabolic control of the initial slope of the *A–C*_i_ response to reside with PEPC activity and the plateau shared by Rubisco and PPDK, with Rubisco accounting for 70% of the control (25). This indicates that upregulation of Rubsico would increase *A*_sat_ in today’s elevated [CO_2_] atmosphere.

Form I Rubisco, found in plants, is made up from eight small subunits (SS) encoded by the nuclear gene family *RbcS* and eight large subunits (LS) encoded by the chloroplast gene *rbcL*. Previously, transgenic increase in Rubisco content, by overexpression of the Rubisco small-subunit (*RbcS*) and Rubisco accumulation factor 1 (*Raf1*) genes, increased *A*_sat_ and plant growth of maize under optimal and chilling growth conditions (26, 27). However, this was confined to a single hybrid genotype of maize grown in controlled environments. To test the hypothesis that this improvement would be more generally possible across this key economic clade of crops and result in increased productivity in the field and greenhouse, Rubisco was upregulated in both sorghum and sugarcane.

## Results

### Transgenic Upregulation of *RbcS* and *Raf1* Genes Increases Rubisco Abundance in Sorghum and Sugarcane.

**Fig. 1.**
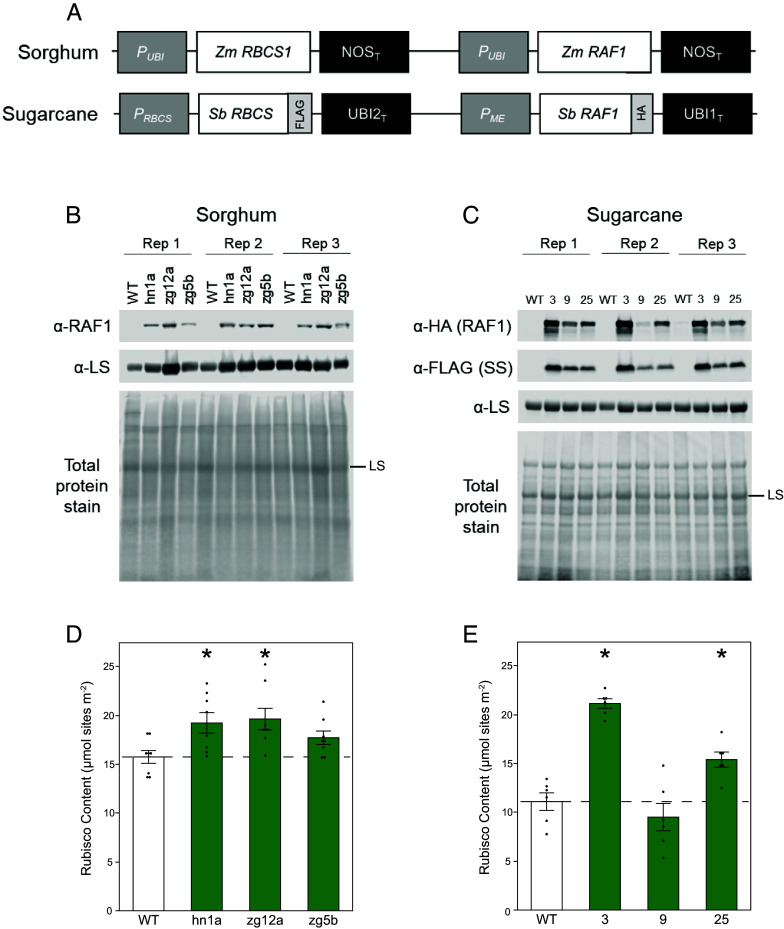
Sorghum and sugarcane transformation constructs, protein expression, and analysis of Rubisco content. (*A*) Transgenes designed to upregulate Rubisco small subunit (*RbcS*) and Rubisco accumulation factor 1 (*Raf1*). The transgenes were stably transformed into sorghum (*Top*) and sugarcane (*Bottom*). The sugarcane construct contains FLAG and HA epitope tags. P, promoter; T, terminator, Sb, *S. bicolor*; Zm, *Zea mays*; UBI, ubiquitin; NOS, nopaline synthase; ME, NADP-malic enzyme; HA, hemagglutinin. (*B*) Total soluble protein isolated on a leaf area basis from sorghum and (*C*) sugarcane, and analyzed by immunoblot. Three replicate plants (Rep) of each of the three transgenic events and of the wild-type (WT) control were probed with antibodies indicated to the left. -LS marks the Rubisco large subunit band. (*D*) Average Rubisco content per unit leaf area quantified by ^14^C–CABP binding in 6-wk-old sorghum (n = 8), and (*E*) 9-wk-old sugarcane (n = 6) grown in the greenhouse. Values are shown as the mean ± SEM. Asterisks indicate significant differences between WT and the transgenic line (*P < 0.05); one-way ANOVA, Dunnett’s post hoc test.

Two transgenes, each designed to upregulate *RbcS* and *Raf1*, were stably transformed into sorghum and sugarcane (Fig. 1*A*). The construct transformed into sorghum contains constitutive promoters, while the one transformed into sugarcane contains bundle sheath–specific promoters as well as C-terminal epitope tags FLAG and HA. For sorghum, 9 transgenic events were received and 7 determined to be single insertion lines. Transgene expression was confirmed by copy number analysis and western blot (*SI Appendix*, Fig. S1*A*). T1 plants from 7 events were screened for increased CO_2_ assimilation (*A*) and Rubisco activity (*V*_c,max_) using gas exchange (*SI Appendix*, Fig. S1 *B*–*G*). For sugarcane, 49 transgenic events were received and analyzed via western blot and a subset of events screened for improved *A* and *V*_c,max_ (*SI Appendix*, Fig. S2). The top three performing events from each species based on gas exchange and protein data were then characterized along with a wild-type (WT), from which the transformants were derived, propagated alongside these. In sorghum, homozygous plants were obtained from each single insertion event (hn1a, zg12a, and zg5b) at T2. Commercial sugarcane is clonally propagated, and the three events (3, 9, and 25) were effectively clones of the T0 events.

qPCR confirmed transgene expression in sorghum lines characterized in both the greenhouse and field. All three transgenic events showed high levels of *ZmRbcS* and *ZmRaf1* expression, with none in the WT (*SI Appendix*, Fig. S3 *A*–*D*). Immunoblots similarly showed high RAF1 protein expression in the transgenic lines relative to WT (Fig. 1*B*). Epitope tags showed expression of sorghum SS and RAF1 proteins in the sugarcane transformants (Fig. 1*C*).

Rubisco content, measured by the ^14^C–CABP binding assay, was significantly increased by 13 to 25% in sorghum, and by 90% and 39% in sugarcane events 3 and 25 respectively (Fig. 1 *D* and *E*). No significant increase was measured in event 9, even though immunoblots showed the presence of the recombinant SS and RAF1, although the latter at lower levels than in events 3 and 25 (Fig. 1*C*). There were no differences in total soluble protein between WT and transgenic plants in either sorghum or sugarcane (*SI Appendix*, Table S3).

### Co-Upregulation of RBCS and RAF1 Is Correlated With Increased *V*_c,max_, CO_2_ Assimilation, and Photosynthetic Light Induction in Sorghum.

**Fig. 2.**
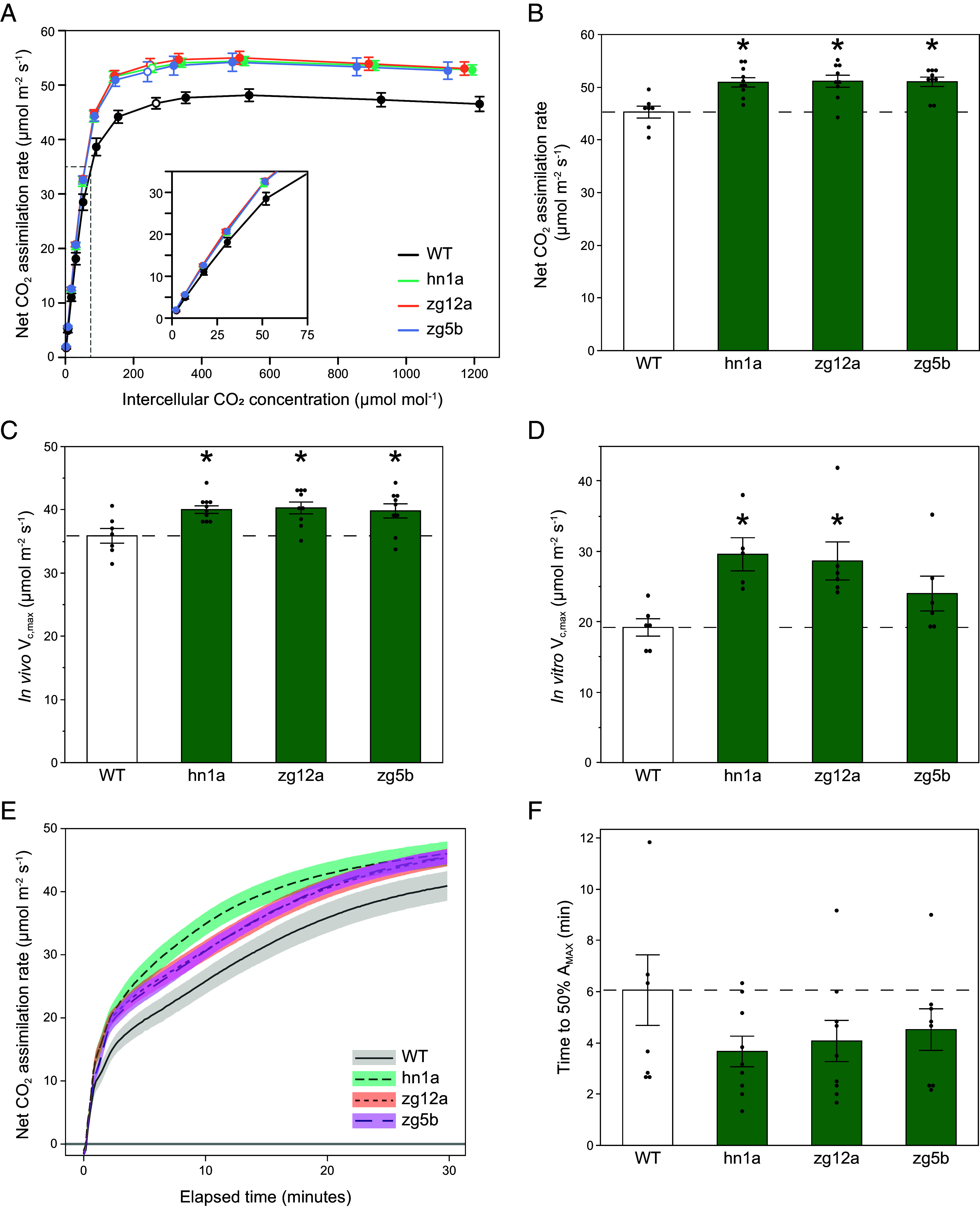
CO_2_ response curves and photosynthetic induction to light of greenhouse-grown sorghum plants. (*A*) Response of light-saturated CO_2_ assimilation rates (*A*_sat_) to intercellular [CO_2_] (C_i_). Measurements were made under the following conditions: light intensity of 1,800 µmol m^−2^ s^−1^, leaf temperature of 28 °C, and VPD 1.5 kPa. Measurement cuvette [CO_2_] were varied from 10 to 1,500 µmol mol^−1^ CO_2._ Open points represent the measured value closest to the operating *C*_i_ (i.e the *C*i where ambient CO_2_ was 420 µmol mol^−1^). (*B*) *A*_sat_ measured at 400 µmol mol^−1^ CO_2._ (*C*) Maximum in vivo Rubisco carboxylation rate (*V*_c,max_) at 25 °C estimated from response curves in panel a (n = 7 to 10). (*D*) Maximum in vitro Rubisco carboxylation rate (*V*_c,max_) at 25 °C (n = 5 to 6). (*E*) Average CO_2_ assimilation during the first 180 seconds of induction. (*F*) Time for *A* to reach 50% of *A*_max,_ where *A*_max_ is the average *A* from 25 to 30 min of illumination. Measurements were made on 5-wk-old plants. Values are shown as the mean ± SEM (n = 7 to 10). Asterisks indicate significant differences between WT and the transgenic line (*P < 0.05); one-way ANOVA, Dunnett’s post hoc test.

*A–C*_i_ responses showed that upregulated RBCS and RAF1 significantly increased assimilation rates above a *C*_i_ of ~100 µmol mol^−1^, but not below (Fig. 2*A*). Maximum in vivo Rubisco carboxylation rates (V_c,max_) at 25 °C, estimated from the *A–C*_i_ response, were significantly increased by 12% in the transgenic lines (Fig. 2*C*), while maximum PEPC carboxylation rates (V_p,max_) though slightly higher than WT did not differ significantly (Table 1). In vitro V_c,max_ rates were increased by ~40% in the transgenic lines (Fig. 2*D*). *A*_sat_ measured at the current atmospheric [CO_2_] also showed significant increases in the transgenic lines (Fig. 2*B*). *g*_sw_ was similarly significantly increased but this cannot account for increases in *A*_sat_ because no differences in *C*_i_ were observed between genotypes (Table 1). The effective quantum yield of PSII (Φ_PSII_), measured by modulated chlorophyll fluorescence, was increased by 12% in the transformed plants in parallel with CO_2_ assimilation (Table 1). Rubisco activation state, the fraction of Rubisco active sites that are catalytically active and therefore contributing to carboxylation, was not significantly different between lines (Table 1).

**Table 1.** Summary of biochemical and leaf gas exchange measurements from greenhouse-grown sorghum and sugarcane

Parameter	WT	RBCS-RAF1 Hn1a	RBCS-RAF1 Zg12a	RBCS-RAF1 Zg5b	Sample number (*N*)
Sorghum—greenhouse					
Initial rubisco activity (µmol m^−2^ s^−1^)	13.48 ± 1.16	**19.00 ±1.45**	**22.28 ± 1.61**	17.60 ± 1.20	5 to 6
Rubisco activation state (%)	71.41 ± 6.99	64.89 ± 4.47	79.21 ± 5.31	75.38 ± 5.79	5 to 6
ΦPSII	0.299 ± 0.002	**0.336 ± 0.007**	**0.331 ± 0.009**	**0.338 ± 0.006**	7 to 10
*V*_p,max_ (µmol m^−2^ s^−1^)	80.32 ± 7.38	88.33 ± 3.19	88.05 ± 3.59	89.99 ± 2.93	7 to 10
*C*_i_ (µmol mol^−1^)	153.53 ± 2.36	142.53 ± 2.18	144.62 ± 3.55	144.50 ± 4.95	7 to 10
*g_sw_*(mol m^−2^ s^−1^)	0.411 ± 0.010	**0.463 ± 0.009**	**0.472 ± 0.012**	**0.472 ± 0.014**	7 to 10
$\overset{-}{A}$*_180_* (µmol m^−2^ s^−1^)	10.31 ± 1.17	**14.25 ± 1.10**	**13.99 ± 0.90**	12.83 ± 0.78	8 to 9
Parameter	WT	RBCS-RAF1 3	RBCS-RAF1 9	RBCS-RAF1 25	Sample number (*N*)
Sugarcane—greenhouse					
Initial rubisco activity (µmol m^−2^ s^−1^)	15.89 ± 0.86	**23.84 ± 2.84**	**23.74 ± 1.58**	20.35 ± 1.81	6
Rubisco activation state (%)	82.30 ± 4.38	81.86 ± 4.54	87.22 ± 3.10	79.40 ± 7.51	6
ΦPSII	0.297 ± 0.012	0.319 ± 0.008	0.300 ± 0.009	0.326 ± 0.009	10
*V*_p,max_ (µmol m^−2^ s^−1^)	68.82 ± 4.55	85.70 ± 6.97	68.99 ± 3.02	79.93 ± 4.76	9 to 10
*C*_i_ (µmol mol^−1^)	116.48 ± 8.61	113.76 ± 6.29	121.17 ± 4.47	118.55 ± 3.27	9 to 10
*g_sw_*(mol m^−2^ s^−1^)	0.252 ± 0.012	0.282 ± 0.006	0.282 ± 0.007	**0.305 ± 0.015**	9 to 10
$\overset{-}{A}$*_300_* (µmol m^−2^ s^−1^)	11.05 ± 2.26	17.78 ± 2.08	5.62 ± 1.95	15.38 ± 1.77	7 to 8
Time to 50% *A_MAX_* (min)	3.36 ± 0.38	3.05 ± 0.27	**9.27 ± 1.62**	3.35 ± 0.26	6 to 8

Values are shown as the mean ± SEM. Values significantly different from WT at *P* < 0.05 are in bold. One-way ANOVA, Dunnett’s post hoc test.

To identify whether increased Rubisco would be beneficial to the activation of photosynthesis on shade to sun transition, induction of *A* over the first 30 min of transfer to high-light (PPFD = 1,800 µmol m^−2^ s^−1^) was measured (Fig. 2*E*). CO_2_ assimilation in the transgenic lines was significantly higher than WT after 3 min of illumination following dark acclimation (Table 1) and remained higher for the remaining 27 min of measurement (Fig. 2*E*). Induction curves, normalized to the average *A* value over the last 5 min of illumination (*A*_max_) were used to assess the speed of photosynthetic activation (*SI Appendix*, Fig. S4*A*). On average, the transgenics took 4 min to reach 50% maximum CO_2_ assimilation rates (*A*_max_), compared to 6 min for WT (Fig. 2*F*). The transgenics were significantly taller and had more leaves than WT in the greenhouse (*SI Appendix*, Fig. S5). Seed from these plants was collected for the field trial.

### RBCS-RAF1 Transgenic Sorghum Plants have Increased Photosynthesis and Biomass Under Field Conditions.

**Fig. 3.**
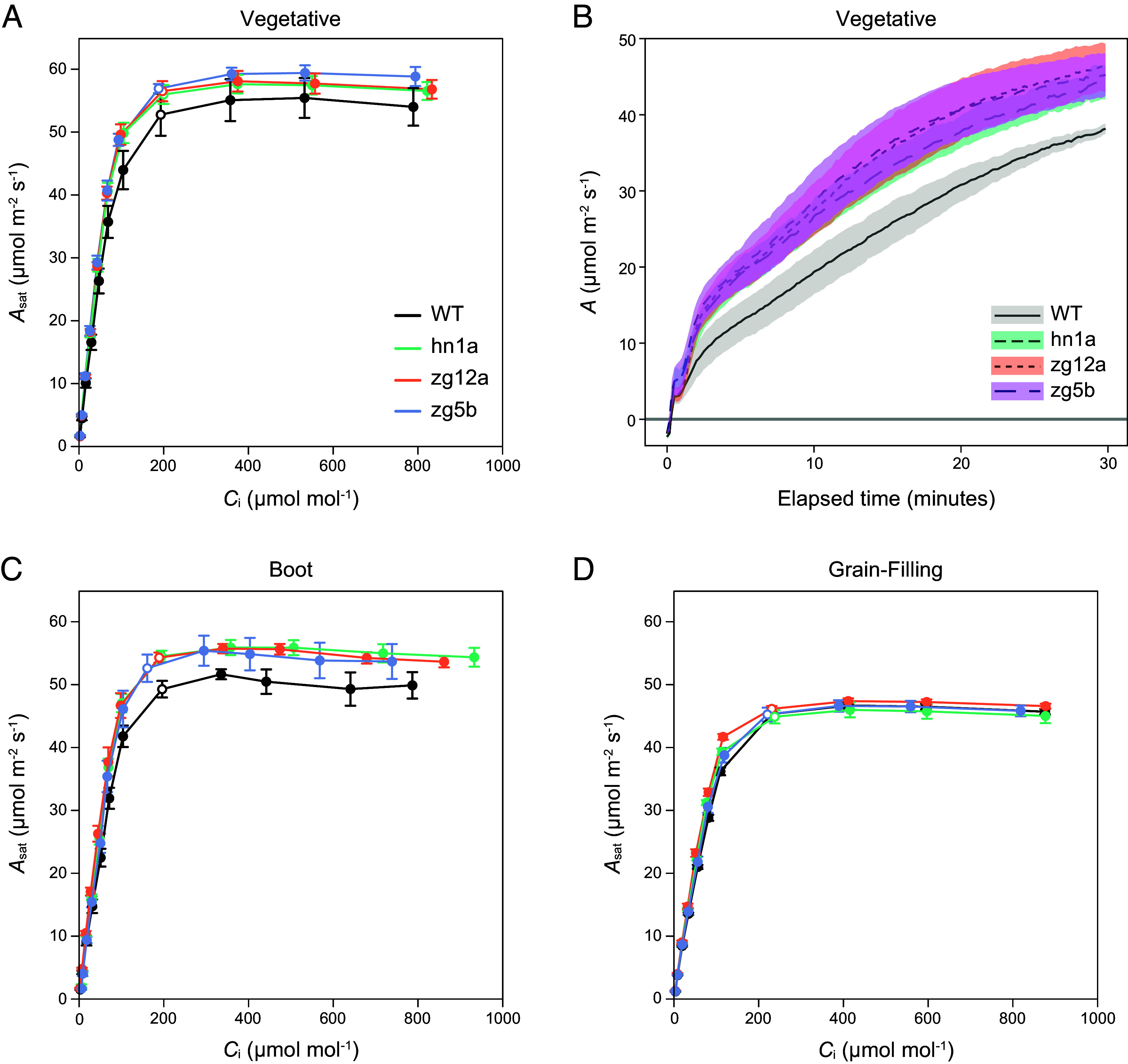
CO_2_ response curves and photosynthetic induction to light of field-grown sorghum plants. (*A*) Response of light-saturated CO_2_ assimilation rates (*A*_sat_) to intercellular [CO_2_] (*C*_i_) and (*B*) induction of *A* during the first 30 min of illumination at PPFD 1,800 µmol m^−2^ s^−1^ of sorghum plants in the vegetative growth stage. (*C*) *A*_sat_*–C_i_* response of sorghum plants in the boot and (*D*) grain-filling growth stages. Open symbols on each curve show the measured value closest to the operating C_i_ (where ambient CO_2_ is 420 µmol mol^−1^). Values are shown as the mean ± SEM (n = 4 to 6).

A field trial in summer 2023 evaluated whether gains observed in the greenhouse could be reproduced in a replicated field trial of 4-row plots. A randomized complete block design was used with 6 replicate plots for each of the three RBCS-RAF1 transgenic events and WT (*SI Appendix*, Fig. S6*A*). *A–C*_i_ responses and light induction curves measured during the vegetative growth stage were consistent with the greenhouse results. *V*_c,max_, was again increased, with no significant increase in the initial slope of the response, and light induction of *A* was faster (Fig. 3). These increases were also observed at boot stage but were absent during grain-filling (Fig. 3 *C* and *D*). Bundle sheath leakiness (*Ф*), the rate that CO_2_ not fixed by Rubisco leaks from bundle sheath back to mesophyll, was measured by combining gas exchange with carbon isotope discrimination during the vegetative growth stage. In concert with increased *V*_c,max_, *Ф* was decreased by about 18% across all three transgenic lines; however, these decreases were not statistically significant (*SI Appendix*, Fig. S7).

**Fig. 4.**
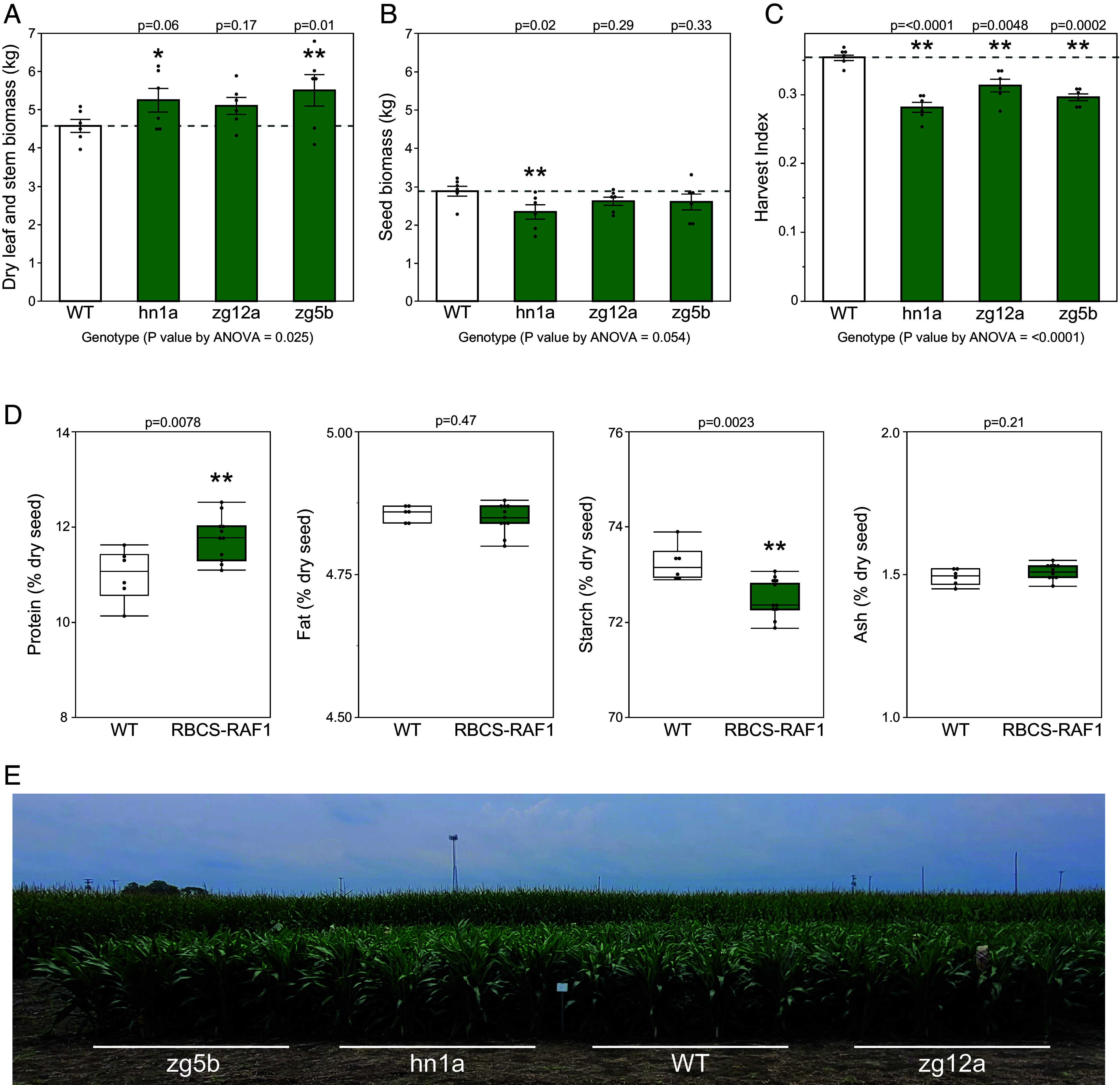
Plant growth traits in field-grown sorghum plants. (*A*) Average dry leaf and stem biomass per plot and (*B*) average seed biomass per plot from field-grown sorghum plants. (*C*) Harvest index (seed mass/total shoot mass) per plot. Values are shown as the mean ± SEM (n = 6 plots). Asterisks indicate significant differences between WT and the transgenic line (**P < 0.05, *P < 0.1); one-way ANOVA, Dunnett’s post hoc test. *P* values above each bar from Dunnett’s post hoc test. (*D*) Percent protein, fat, starch, and ash content of whole dry sorghum seeds (WT n = 6 plots, RBCS-RAF1 n = 11 plots). **P < 0.05; pooled *t* test. (*E*) Side view of 4 of the 24 four-row plots of the four sorghum genotypes tested at boot stage in the field in Urbana, Illinois summer 2023 (*SI Appendix*, Fig. S6*A* for full experimental design).

The field-grown transgenic sorghum plants were also more vigorous than the WT, as evidenced by a significant 15.5% increase in vegetative dry biomass at harvest (Fig. 4*A*). However, there was no increase in seed biomass, which corresponded to a significant decrease in harvest index (seed mass/total plant mass) compared to the WT (Fig. 4 *B* and *C*). Seed composition was analyzed using NIR and showed increased protein and decreased starch content in the transgenic lines, with no change in fat or ash content (Fig. 4*D*). Number of panicles per plot, 100 seed weight, and average seed weight per panicle did not show any clear trends; however, panicle emergence was delayed by 3 to 6 d in the transgenic plants (*SI Appendix*, Table S2). Leaf mass, chlorophyll content, N and C contents, on a leaf area basis, did not differ between the WT and transgenic plants (*SI Appendix*, Table S3).

### Co-Upregulation of RBCS and RAF1 in Sugarcane Increases Photosynthetic Efficiency and Plant Productivity.

**Fig. 5.**
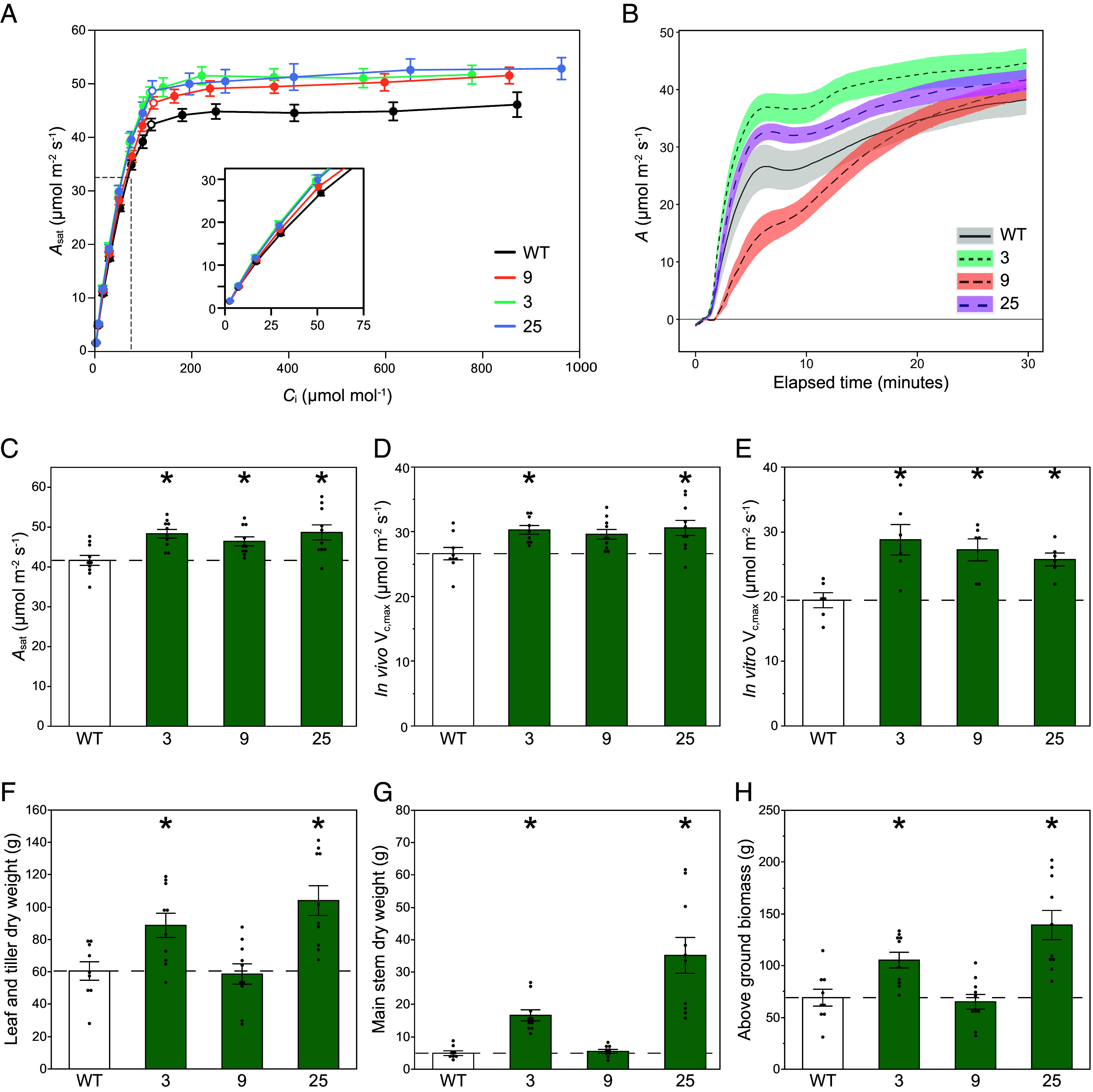
CO_2_ response curves and plant growth traits of greenhouse-grown sugarcane plants. (*A*) Response of light-saturated CO_2_ assimilation rates (*A*_sat_) to intercellular [CO_2_] (*C*_i_). Measurements were at light intensity of 1,800 µmol m^−2^ s^−1^, leaf temperature 30 °C, and 65% humidity. Measurement cuvette [CO_2_] was varied from 10 to 1,500 µmol mol^−1^_._ Open symbols show the measured value closest to the operating C_i_ (where ambient [CO_2_] was 420 µmol mol^−1^). (B) Induction of *A* during the first 30 min of illumination at PPFD 1,800 µmol m^−2^ s^−1^. (*C*) *A*_sat_ at 450 µmol mol^−1^ CO_2_ from panel *A*. (*D*) Maximum Rubisco carboxylation rate in vivo (*V*_c,max_) at 25 °C estimated from response curves in panel a (n = 8 to 10). (*E*) Maximum in vitro Rubisco carboxylation rate (*V*_c,max_) at 25 °C (n = 6). (*F*) Leaf and tiller, (*G*) main stem, and (*H*) above-ground total dry biomass of greenhouse-grown sugarcane plants. Values are shown as the mean ± SEM (n = 8 to 10). Asterisks indicate significant differences between WT and the transgenic lines (*P < 0.05); one-way ANOVA, Dunnett’s post hoc test.

Measurements were made on 8-wk-old greenhouse-grown sugarcane plants. As in sorghum, *A–C*_i_ responses showed significant increases in in vivo Rubisco activity (*V*_c,max_) with no significant change in the initial slope (*V*_p,max_, Fig. 5 *A* and *D* and Table 1). Similarly, *A*_sat_ and in vitro *V*_c,max_ were significantly increased at ambient atmospheric [CO_2_] in the transgenics (Fig. 5 *C* and *E*). There were no significant differences in Rubisco activation state, *g*_sw_ or *C*_i_: However, there was a marginal increase in Φ_PSII_ in events 3 and 25 (Table 1). Assimilation of CO_2_ was greater over the 30 min light induction, in events 3 and 25, but not event 9 potentially due to a pleotropic effect related to the location of the transgene insertion into the genome (Fig. 5*B* and *SI Appendix*, Fig. S8).

Transgenic events 3 and 25 showed significant increases in main stem, and tiller and leaf dry mass, amounting to total shoot dry mass increases of 37% and 81% respectively, but with no increase in event 9 (Fig. 5 *F*–*H*). Significant increases in plant height, stem width, and leaf number were also observed in events 3 and 25 compared to WT (*SI Appendix*, Fig. S9). Leaf chlorophyll and mass per unit area did not differ between genotypes (*SI Appendix*, Table S3).

## Discussion

Theoretical and experimental analyses have long suggested Rubisco to be the major limitation of light-saturated photosynthesis in both C_3_ and C_4_ species (7, 18–20, 28). Consistent with this hypothesis, transgenic upregulation of Rubisco content significantly increased Rubisco carboxylation rates (*V*_cmax_) and light-saturated leaf CO_2_ assimilation rates (*A*_sat_) in both sorghum and sugarcane (Figs. 1 *D*–*E*, 2 *A*–*D*, 3 *A*–*C*, and 5 *A* and *C*–*E*). Although Rubisco or electron transport can limit assimilation in the plateau of a C_4_ *A*–*C_i_* curve (7, 29), the agreement between in vivo and in vitro *V_cmax_* estimates is consistent with primarily Rubisco-limited assimilation at high light (Figs. 2 *A*, *C*, and *D* and 5 *A* and *D*–*E*). The speed of light induction of CO_2_ assimilation was also increased (Figs. 2 *E* and *F* and 3*B*). This has critical importance to increasing photosynthetic efficiency under the fluctuating light conditions of crop canopies in the field (30, 31). Also consistent with limitation by Rubisco, bundle sheath leakiness was decreased, although not significantly (*SI Appendix*, Fig. S7). Finally, and in concert with these increases in photosynthetic efficiency, a field trial of sorghum and a greenhouse trial of sugarcane showed significant increases in productivity relative to the wild-type from which the transgenics were derived (Figs. 4 and 5).

Rubisco is, by far, the most abundant single protein in leaves, constituting up to 50% of soluble protein in C_3_ plants and 20 to 30% in C_4_ (32). These high amounts are attributed to the need to offset the exceptionally low catalytic rate of carboxylation (*k*_cat_) of Rubisco relative to other enzymes. Its low *k*_cat_ is in turn considered in part a consequence of high specificity for CO_2_ relative to the alternative substrate of O_2_ (33). Sage & McKown (34) noted the much lower available tissue volume that could house Rubisco in C_4_ leaves, where it is functionally confined to the bundle sheath, suggesting this as a constraint. This is less than half the volume available in C_3_ leaves. However, 3D microscopic analysis of the bundle sheath chloroplast stromal volume in *Miscanthus* x *giganteus* suggested capacity for a doubling of actual Rubisco content (35). *Miscanthus* is closely related to sorghum and sugarcane, and within the same subtribe, Saccharinae (36). This observation is consistent with the observed increases in Rubisco content of 25% in sorghum and up to 90% in sugarcane (Fig. 1 *D* and *E*). The much higher level in sugarcane may reflect that this was achieved by biolistic transformation, which could have resulted in multiple insertions of the transgenes, while the three sorghum events were single insertion events.

Given that increased Rubisco production is possible and beneficial, why have natural selection and breeding not achieved this? One explanation is that Rubisco might only have become a limitation very recently, due to rising atmospheric [CO_2_]. On March 16, 2024, atmospheric [CO_2_] was recorded at 428 μmol mol^−1^ (21), which is almost double the average atmospheric [CO_2_] of the past 40,000 y (22, 37). Theory suggests that under the conditions in which our crop ancestors evolved, Rubisco would not be a limitation and therefore selection pressure would have been lacking, with the result that there may be little genetic variation for natural or breeder selection as [CO_2_] rises. Indeed given the large nitrogen cost of Rubisco, historically, selection pressure was likely against any increase. It should be noted that half of the increase in [CO_2_] has occurred in the last 50 y, with [CO_2_] rising from 323 μmol mol^−1^ to 428 μmol mol^−1^ between 1974 and 2024 (21). By reference to the open symbols in Fig. 3, it is seen that at today’s atmospheric [CO_2_] *A*_sat_ in the field-grown sorghum is controlled by the plateau of the *A*–*C*_i_ response, i.e. predominantly by Rubisco activity. However, if it is assumed that *C*_i_/*C*_a_ has been conserved, then at the atmospheric [CO_2_] in 1974, *C*_i_ would equal 129 μmol mol^−1^. By reference to Fig. 3, *A*_sat_ would then be mostly controlled by the initial slope of the *A*–*C*_i_ response, i.e. PEP carboxylase activity (*V*_p,max_), and insensitive to increase in Rubisco activity. The period in which Rubisco has become limiting is therefore very short in terms of reproductive cycles. As atmospheric [CO_2_] continues to rise, Rubisco limitations will increase, and elevation of Rubisco will be an important means to contribute toward future-proofing and advancing C_4_ crop productivity. An increase in *A*_sat_ was achieved here by only upregulating expression of *RbcS* and *Raf1,* which infers three factors important for future manipulations. First, for Rubisco to be increased, both the large (LS) and small (SS) subunits must be increased. That only upregulation of *RbcS* was needed, is consistent with the observations that in tobacco *rbcL* is subject to assembly-dependent translational regulation, and so controlled by the supply of SS (38) or in rice the supply of SS upregulates *rbcL* transcript levels (39). Further studies are required to determine which mechanism is occurring in sorghum and sugarcane. Second, although all three transgenic sugarcane events showed expression of the *Sb* SS protein, in event 9 this did not translate into improved productivity (Figs. 1 *C*–*E* and 5 *F*–*H*). Expression of *Sb* RAF1 was lowest in this line, suggesting the importance of effective expression of RAF1 with SS (Fig. 1*C*). Third, there must either be 1) overcapacity in the C_4_ cycle, 2) the C_4_ cycle increases capacity in concert with increased Rubisco activity, or 3) both occur. The fact that bundle sheath leakiness of CO_2_ decreased and *V*_p,max_ slightly increased with increased Rubisco is consistent with hypothesis 3 (24).

Similar to results reported in maize (26), increases in Rubisco content corresponded to increases in Rubisco enzyme activity, resulting in higher photosynthetic rates and larger biomass. Activation of Rubisco requires the chaperone protein Rubisco activase (RCA). In contrast to previous studies in maize and rice (26, 40), no decrease in the fraction of active Rubisco was observed in the transgenic plants, indicating that sorghum and sugarcane must have had sufficient RCA to support the activation of all their additional Rubisco (Table 1). In these previous studies, increased Rubisco content was achieved by overexpressing endogenous *RbcS* with or without *Raf1*. Here, *RbcS* and *Raf1* genes from maize and sorghum were expressed in sorghum and sugarcane respectively, so the transgenic plants are assembling some chimeric Rubiscos composed of the foreign transgenic SS and endogenous LS. Maize, sorghum, and sugarcane have been reported to have very similar *k_cat_* (41, 42), implying that the increases in Rubisco activity reported here are solely due to increased Rubisco active sites and not changes in Rubisco kinetic properties. In addition, the FLAG tag fused to *RbcS* in the sugarcane construct did not appear to interfere with Rubisco activity (Fig. 5*E*).

In dense modern crop canopies, only the uppermost leaves might experience light-saturated conditions. Most leaves will experience frequent light fluctuations, or sunflecking, due to the passage of clouds, shadowing by overlying leaves as solar angle changes and wind-driven movements (30, 31). The speed with which photosynthetic efficiency can adjust to light fluctuations has been shown to have a profound effect on whole crop daily CO_2_ assimilation and productivity (43, 44). A key factor here is the speed of induction of *A* on transfer of a leaf from shade to sun. Modeling and experimental studies have shown the speed at which Rubisco activity increases to be critical to induction in both C_3_ and C_4_ crops (23, 45, 46). Here, increased Rubisco content coincided with more total CO_2_ assimilation over the 30 min of induction in all three sorghum events and two of the sugarcane events (Figs. 2*E*, 3*B*, and 5*B*). Speed of induction, measured as time to 50% *A*_max_, was also increased in the transgenic sorghum lines but not in sugarcane (Fig. 2*F* and Table 1). This is consistent with computational results from Wang et al. (23), which suggest that Rubisco activation is the main limiting factor in the speed of photosynthetic induction in sorghum, while the speed of stomatal opening and the concentration of the PPDK regulatory protein exert more control in sugarcane.

In the greenhouse, significant increases in growth of both sorghum and sugarcane were observed at an individual plant level (Fig. 5 *F*–*H* and *SI Appendix*, Fig. S5). Importantly, and consistent with increased CO_2_ assimilation rates during vegetative and boot stages, leaf and stem biomass was significantly increased by an average of 15.5% in the RBCS-RAF1 sorghum field plots relative to the WT controls, a desired characteristic for C_4_ biomass crops (Fig. 4*A*). However, grain yield per plot did not increase, due to a significant decrease in harvest index (Fig. 4*C*). Although sorghum cultivar RTx430 has become the standard for transformation and experimental analyses, it is a 40-y-old inbred line (47–49) and might be sink-limited. There is similar evidence for sink limitations in a 25-y-old hybrid cultivar; when sorghum was grown under well-watered conditions under open-air CO_2_ enrichment using FACE technology, photosynthetic capacity and photosynthetic rates were increased (50–52). Yet, as in our experiment, stover increased while harvest index declined by 7% (53). Sink limitation at grain filling results in accumulation of nonstructural carbohydrates in the source tissues, causing downregulation of photosynthetic genes, notably *RbcS* (54). The loss of in vivo Rubisco activity (*V*_c,max_) during grain filling evident in Fig. 3*D* is consistent with sink limitation. Alternatively, grain yields may not have increased due to alternative factors such as delayed panicle emergence or decreased transgene expression. It is also important to note that Rubisco levels increased without an associated change in total soluble protein, LMA, or leaf N content (Fig. 1 and *SI Appendix*, Table S3). This suggests that more leaf N is invested into Rubisco in the transgenic plants and therefore less N allocated to other leaf proteins. Although there was no negative impact on photosynthesis, altered N use may affect other processes including grain filling. Testing whether greater grain yields can be obtained will require the production of hybrids and/or transformation of current elite cultivars. Upregulation of Rubisco has been achieved here, as a test of the concept that more Rubisco would translate to more efficient photosynthesis and productivity in today’s atmosphere, by addition of extra copies of *RbcS* and *Raf1.* Alternatively, a future approach for upregulating Rubisco could be using DNA editing to mutate repressive elements in the promoter regions of the native *RbcS* and *Raf1* genes.

Despite significant breeding efforts, the average yield of sorghum has not changed over 30 y, nor has the yield of sugarcane in the United States in the last 20 y (*SI Appendix*, Fig. S10 *A* and *B*). This might reflect increasingly adverse weather conditions. However, even in the United States where it might be assumed that favorable agronomy would allow the yield potential to be realized, there is little evidence of yield improvement since 1991, even in the most favorable years (*SI Appendix*, Fig. S10*A*). Therefore, if the photosynthetic and productivity improvements found here by upregulating Rubisco translate into parallel yield improvements in elite cultivars and hybrids, this could be very useful for integration into breeding programs. This successful test-of-concept suggests multilocation and/or multiyear replicated yield trials of Rubisco upregulation in elite material as the next proving step. Given the great value and global significance of this key economic clade of crops both for food and biomass, the results here indicate an important opportunity to achieve increased productivity under rising atmospheric [CO_2_].

## Methods

### Sorghum Plasmid Assembly and Transformation.

A 3.6 kb fragment containing the *Raf1* expression cassette regulated by the maize ubiquitin promoter was isolated from the plasmid pPTN1063 (26) by digesting the vector with *Kpn*I and *Hind*III. The fragment was then blunted with T4 DNA polymerase. Plasmid pPTN438 (38) (containing the *RbcS* expression cassette) was linearized via *Kpn*I digestion and blunted with T4 DNA polymerase. The pPTN1063 fragment and linearized pPTN438 plasmid were then ligated resulting in the final binary vector pPTN1589. Sorghum inbred RTx430 was transformed with pPTN1589 using agrobacterium as previously described (55).

### Sugarcane Plasmid Assembly.

Recombinant DNA constructs were assembled with Golden Gate cloning to highly express Rubisco small subunit (*RbcS*) and Rubisco accumulation factor 1 (*Raf1*) from *S. bicolor* in sugarcane. The open reading frame (ORF) of *SbRbcS* (Sobic.005G042000) and ORF of *SbRaf1* (Sobic.006G269400) were synthesized with 1X FLAG tag and 6X HA tag, respectively (GenScript, Piscataway, NJ). *SbRbcS*-FLAG was subcloned under transcriptional control of the maize RBCS promoter + 5’UTR and the *Panicum virgatum* UBI2 (PvUBI2) terminator. The synthesized *SbRaf1*-HA was subcloned under transcriptional control of the maize NADP-ME promoter and *Panicum virgatum* UBI1 (PvUBI1) terminator. The selectable marker gene *npt*II was subcloned under transcriptional control of the ubiquitin promoter from *Zea mays* and SbHSP16.9 terminator from *S. bicolor*. The three expression cassettes were subcloned into a L2 construct downstream of an RB7 matrix attachment region (MAR) and upstream of TM2 MAR from *Nicotiana tabacum* serving as insulators and confirmed by Sanger sequencing. The minimal expression construct flanked by MARs was excised from the plasmid backbone using the *I-Sce*1 restriction enzyme and gel purified prior to biolistic transformation into sugarcane.

### Sorghum Transgene Copy Number Analysis.

Transgene copy number was measured using digital droplet PCR (ddPCR) following the protocols from Głowacka et al. (56), with slight modifications. *SI Appendix*, Table S1 for primers used (57). Sorghum genomic DNA was digested overnight with HindIII. A 20 μL ddPCR reaction mix contained 20 ng of the digested DNA, 10 μL QX200 ddPCR EvaGreen Supermix (186-4034, BioRad, Hercules, CA) and primers at a final concentration of 100 nM. Droplets were generated using a QX200 droplet generator (186-4002, Bio-Rad) by combining 20 μL of the reaction mix with 70 μL of EvaGreen droplet generation oil (186-4005, Bio-Rad). The PCR was conducted in a deep-well thermal cycler (C1000 Touch, 185-1196, Bio-Rad) with the following conditions: 95 °C for 5 min, 40 cycles of 95 °C for 30 seconds, and 59 °C for 1 min, then 4 °C for 5 min and 90 °C for 5 min, with a ramp rate of 2.5 °C/second at each step. Immediately after the PCR, measurements were taken using a QX200 droplet reader (186-4003, Bio-Rad).

### Generation and PCR Confirmation of Transgenic Sugarcane Plants.

The linearized, minimal construct containing *SbRbcS*, *SbRaf1,* and NPTII cassettes flanked by matrix attachment regions was introduced into sugarcane cv. CPCL02-0926 by biolistic gene transfer as described previously (58). Briefly, cross-sections of sugarcane leaf whorls above the shoot apical meristem were cultured on a modified Murashige & Skoog medium (MS) with B5 vitamins (PhytoTech Labs, KS) supplemented with 3 mg/L of 2,4-Dichloro phenoxy acetic acid (PhytoTech Labs) and maintained at 28 °C in the dark for induction of embryogenic callus. For biolistic gene transfer, the minimal expression cassette was coated onto gold particles and introduced into embryogenic calli using biolistic PDS-1000/He delivery system (Bio-Rad) as described previously (59). Following 4 wk of selection of transformed calli on the medium containing the antibiotic Geneticin®, calli were subcultured onto regeneration media (MS media with B5 vitamins, supplemented with 1.86 mg/L of α-Naphthaleneacetic acid/NAA (PhytoTech Labs) and 0.09 mg/L of 6-Benzylaminopurine/BAP (PhytoTech Labs)) and maintained at 28 °C incubator with 16/8 h light (100 µmol m^−2^ s^−1^) and dark cycle. Regenerated shoots were transferred to modified MS basal medium (PhytoTech Labs) for shoot elongation and rooting. Rooted plantlets were transferred to soil and acclimatized in a temperature-controlled growth chamber maintained at 28 °C day and 22 °C night temperature with 16/8 h light (400 µmol m^−2^ s^−1^) and dark cycle for growth and further analysis.

For the PCR confirmation of transgenic events, DNA was isolated from 100 mg of leaf tissues using the CTAB method with modifications (60). PCRs were performed on a thermocycler (Applied Biosystems, Foster City, CA) using standard Taq buffer, dNTPs, and hot start Taq polymerase (New England Biolabs Inc, Ipswich, MA). See *SI Appendix*, Table S1 for primers used for PCR amplification. Amplicons were resolved with electrophoresis on a 1.2% agarose gel and visualized under UV transilluminator (Bio-Rad).

### Plant Growth—Greenhouse Conditions.

T2 homozygous *S. bicolor* (inbred RTx430) seeds from 3 independent single insertion events and nontransformed WT seeds from the same harvest date were sowed into plastic potting trays 6 cm × 6 cm × 5.4 cm deep, filled with BM7 growing medium (BM7 All-Purpose Bark Mix, Berger). After about 14 d seedlings were transplanted into 9.5L plastic pots filled with BM7 growing medium supplemented with 44 cm^3^ of 13-13-13 (N-P-K) granulated slow-release fertilizer (Osmocote, ICL-Growing Solutions). Sugarcane (*Saccharum officinarum* cv. CPCL02-0926) plantlets clonally propagated from three independent T0 transformation events and nontransformed WT plants were also grown in the greenhouse. Single node segments from mature stalks were transplanted into 9.5L plastic pots filled with BM7 growing medium supplemented with 44 cm^3^ of 13-13-13 (N-P-K) granulated slow-release fertilizer. Both sorghum and sugarcane were grown under natural illumination (*SI Appendix*, Fig. S11). Lights turned on from 6:30 am to 9:30 pm (15 h days), providing an additional 350 to 450 µmol m^−2^s^−1^ of LED light, and turned off when outside light intensity surpassed 350 W/m^2^ (~800 µmol m^−2^s^−1^). The greenhouse temperature was kept between 25 to 28 °C in the day and 24 to 26 °C at night. Both sorghum and sugarcane plants were fertilized with a rotation of 20-10-20 & 15-5-15 liquid fertilizer (JR Peters INC) at 300 ppm N twice a week. Once a month a MgSO_4_ (100 ppm) and iron (10 ppm) drench was applied (Sprint 138, BASF Corporation).

Sugarcane plants were harvested after 2 mo of growth. Immediately prior to harvesting, leaf number (fully expanded), tiller number, plant height, and stem width were measured. Stem width was measured at the widest point approximately 5 cm above the soil using calipers. Height was measured from the soil to the top of the youngest fully expanded leaf (leaf collar present). Leaf mass per area (LMA) was measured from 3.15 cm^2^ of leaf discs, which were dried until constant weight and weight recorded. Chlorophyll content was measured using a SPAD meter (502, Spectrum Technologies). Harvested material was partitioned into main stem biomass and leaf plus tiller biomass and dried at 60 °C to steady-state weight. Above-ground biomass was calculated as the sum of stem, leaf, and tiller biomass.

### Plant Growth—Field Conditions.

Sorghum seed obtained from the plants in the greenhouse experiment were harvested and used for a field experiment. A randomized complete block design with six blocks was used. Each block contained 16 rows in a North–South direction, made up of four row plots from each of the transgenic lines and WT control (*SI Appendix*, Fig. S6*A*). Prior to planting the ground was worked with a field cultivator (April 27, 2023), and no fertilizer was applied to the soil. Seeds were sowed using a precision planter at a density of 8.6 m^−2^ (Almaco) on June 2nd 2023 on the Crop Sciences Farm of the University of Illinois SoyFACE Experimental Station (40°02’36.0”N 88°13’48.5”W). Weather data were collected with an automatic weather station on the Farm (*SI Appendix*, Fig. S6 *B*–*D*). July 5th Warrior II with Zeon technology (Syngenta) was applied at a moderate rate of 0.07 dm^3^/ha for the control of flea beetles. August 2nd Mustang Maxx (FMC Corporation) was applied at moderate rate of 0.1 dm^3^/ha for the control of aphids.

To avoid any release of pollen, paper bags enclosed the panicles, sealed at the peduncle, prior to anthesis, and remaining until harvest (recorded as panicle emergence date). Plants were harvested by hand on October 4th from the middle two rows of each plot by cutting stems at the ground level. Two plants from either end of each row were discarded to avoid edge effects. Harvested material was partitioned into stem/leaves and panicles with seed. Stem and leaves were dried to a constant weight in custom-made drying ovens at 60 °C and panicles with seed were dried at 30 °C. Dry weights per plot were obtained for both; then, seed was collected using a threshing machine (BT 14-E Belt Thresher, Almaco). The machine was cleaned between each sample to avoid contamination. Total seed weight per plot, and 100 seed weights were then determined. Above-ground biomass was calculated as the sum of stem, leaf, panicle, and seed biomass. Harvest index was calculated as seed biomass divided by total above-ground biomass. Seed weight per panicle was estimated by dividing seed weight per plot by panicle number per plot. Seed protein, starch, fat, and ash content were estimated on a percent basis using a near-infrared reflectance (NIR) analyzer (DA 7250, Perten Instruments). Seeds were placed into the sample dish and analyzed using a built-in program for whole-seed evaluation (“Sorghum-Grain”). Chlorophyll content was measured using a SPAD meter (502, Spectrum Technologies) on August 30th. LMA was measured from 6.06 cm^2^ of leaf tissue collected August 31st_._

### Transcript and Protein Expression.

Prior to RNA and protein extraction tissue was disrupted and homogenized as described previously (61). RNA was extracted from 1.9 cm^2^ of leaf tissue from the youngest fully expanded leaf of greenhouse and field-grown sorghum, using the NucleoSpin RNA Plant Kit (Macherey-Nagel). 700 µL of the lysis buffer was used. RNA quantity and quality were assessed using a NanoDrop (Thermo Fisher Scientific). cDNA was synthesized with Superscript IV VILO Mastermix with ezDNase (Thermo Fisher Scientific). qPCR, primer design, and validation were conducted as described in Salesse-Smith et al 2024 (61), with the exception that an anneal temperature of 60 °C was used for all primer sets. *SI Appendix*, Table S1 for primer sequences. Primer efficiencies were between 100 to 105%. Calibrated Normalized Relative Quantities (CNRQ) were calculated using qBase+ software v.3.2 (CellCarta) based on the expression of two reference genes, SbEIF4α and SbActin11.

Total protein was extracted from 1.9 cm^2^ of leaf tissue from the youngest fully expanded leaf of greenhouse-grown sorghum and sugarcane and field-grown sorghum. Protein extraction and western blots were performed as described previously (61). The following primary antibodies were used: anti-HA (AS12 2220, Agrisera) at a 1:5000 dilution, anti-LS (AS03 037, Agrisera) at a 1:10,000 dilution, anti-FLAG (F7425-.2MG, Sigma-Aldrich) at a 1:5,000 dilution, and anti-RAF1 (custom rabbit antibody, Pacific Immunology) at a 1:1,000 dilution. For the custom antibody, a 23 amino acid immunograde peptide from the maize RAF1 protein (aa 211-233; KGFPRWRGEEGWEAFSKDSPADC) was synthesized and antisera generated by Pacific Immunology. Relative abundance of LS protein was estimated using Empiria Studio Software version 3.2 (Li-COR, Biosciences).

### Rubisco Content, Activity, and Activation Measurements.

Leaf discs were sampled from the youngest fully expanded leaves of greenhouse-grown sorghum (1.9 cm^2^) and sugarcane (2.5 cm^2^) plants, snap-frozen in liquid nitrogen and stored at −80 °C. Samples were taken from the same location gas exchange measurements were made the day after measurements were completed. Soluble protein was extracted in 2 mL of N_2_-sparged extraction buffer: 50 mM EPPS-NaOH pH 8, 1 mM EDTA, 5 mM MgCl_2_, 1% (w/v) PVPP, 5 mM DTT, and 1% (v/v) protease inhibitor cocktail (P9599, Sigma-Aldrich). Frozen leaf discs were ground in ice-cold Ten Broeck homogenizers (7 mL, Corning) containing extraction buffer. Total soluble protein was quantified using the Qubit Protein Assay Kit (Q33211, Life Technologies) on the Qubit 3 Fluorometer (Q33216; Life Technologies). Total Rubisco content was measured by ^14^C–CABP binding as previously described [Ruuska et al. (62); Sharwood et al. (63)]. 100 µL of protein lysate was mixed with an equal volume of Rubisco activation buffer (50 mM EPPS-NaOH pH 8, 1 mM EDTA, 20 mM MgCl_2_, 30 mM NaHCO_3_) and let sit at room temperature. After 20 min, 60 µM of ^14^C-CABP was added to the mixture and left at room temperature for an additional 30 min, and then transferred to ice. 100 µL of the activated Rubisco protein mixture was loaded onto the size-exclusion chromatography columns and fractions containing ^14^C-CABP-Rubisco complexes collected. The amount of ^14^C-CABP was determined by liquid scintillation (Packard Tri-Carb 1900 TR, Canberra Packard Instruments Co). Leaf Rubisco content was calculated as described previously (64).

Rubisco activity was determined at 25 °C by incorporation of ^14^CO_2_ into acid-stable products (65). An extended protocol can be found online: dx.doi.org/10.17504/protocols.io.bf8cjrsw. Sorghum (1.7 cm^2^) and sugarcane (2.26 cm^2^) were collected as indicated above. Soluble protein was extracted in 2 mL of N_2_-sparged extraction buffer: 50 mM Bicine-NaOH pH 8.2, 20 mM MgCl_2_, 1 mM EDTA, 2 mM benzamidine, 5 mM ε-aminocaproic acid, 50 mM 2-mercaptoethanol, 10 mM DTT, 1% (v/v) protease inhibitor cocktail, 1 mM phenylmethylsulfonyl fluoride, and 1% (w/v) polyvinylpolypyrrolidone. Supernatant was immediately used to measure initial and total Rubisco activity, with two technical replicates per measurement. Initial activity was measured by adding 25 µl of protein lysate to assay buffer (100 mM Bicine-NaOH pH 8.2, 20 mM MgCl_2_, 10 mM NaH^14^CO_3_ (18.5 kBq μmol^−1^), 2 mM KH_2_PO_4_, and 0.6 mM RuBP). After 30 s reactions were quenched with 100 µl of 10 M formic acid. For total activity measurements, RuBP was added 3 min after the protein lysate to allow for carbamylation of Rubisco and quenched after 30 s. Reactions were dried at 100 °C, then the residue dissolved in 400 µl deionized water and added to scintillation cocktail for acid-stable ^14^C determination by liquid scintillation. Rubisco activation state was calculated as initial/total activity.

### Leaf Gas Exchange Measurements.

CO_2_ response (*A*–*C*_i_) and light induction curves were measured on the youngest fully expanded leaves using an infrared gas exchange system LI-6800 fitted with the multiphase flash fluorometer and 6 cm^2^ cuvette (6800-01A, LI-COR Environmental). CO_2_ response curves were measured simultaneously with chlorophyll fluorescence and used to assess *A*, *C*_i_, *g*_sw_, ΦPSII, *V*_c,max_, and *V*_p,max_. *A*–*C*_i_ curves were measured in the field-grown sorghum on July 10th, August 4th, and August 29th. Leaves were acclimated to the following conditions: light intensity of 1,800 µmol m^−2^ s^−1^, leaf temperature of 30 °C, CO_2_ reference concentration of 400 µmol mol^−1^, and 65% humidity. Once *A* and *g*_sw_ stabilized, response curves were initiated using the following sequence of reference CO_2_ concentrations: 400, 300, 200, 120, 70, 30, 10, 400, 400, 500, 700, 900, 1,200, and 1,500 µmol mol^−1^. Measurements were logged 3 to 5 min after each new CO_2_ step. *A*–*C*_i_ curves for greenhouse-grown sorghum were measured under the same conditions except for the following changes: A leaf temperature of 28 °C was used and the CO_2_ sequence used concentrations of 600 and 800 µmol mol^−1^ CO_2_ instead of 700 and 900. Sugarcane *A*–*C*_i_ curves used a CO_2_ reference concentration of 450 µmol mol^−1^ and the following sequence of CO_2_: 450, 1,500, 1,250, 1,000, 750, 600, 500, 400, 300, 200, 120, 70, 30, and 10 µmol mol^−1^. *V*_c,max_ and *V*_p,max_ at 25 °C were estimated using the *fit_c4_aci* function from the PhotoGEA R package (66), which fits a mechanistic model to the measured CO_2_ response curves (7). In this method, values of *V*_c,max_, *V*_p,max_, and *R*_L_ are varied to find the best fit of the full equation for enzyme-limited assimilation (Equation 4.21 of von Caemmerer 2000) to all points in each curve (29).

Light induction curves were used to assess average *A* in either the first 180 or 300 s since the light was turned on and time to 50% *A*_max_, which is determined from the normalized induction curves. Leaves were acclimated to the following conditions: light intensity of 0 µmol m^−2^ s^−1^, leaf temperature of 28 °C (greenhouse-grown sorghum) or 30 °C (field-grown sorghum and sugarcane), CO_2_ sample concentration of 400 (sorghum) or 450 µmol mol^−1^ (sugarcane), and 65% humidity. After 30 min in the dark, light was increased to 1,800 µmol m^−2^ s^−1^ and data points logged every 10 s over the course of 30 min. Average assimilation after 300 s of light induction was calculated for sugarcane, while 180 s was used for sorghum due to differences in each species’ overall speed of induction. After gas exchange measurements were completed on the greenhouse-grown sorghum plants, leaf number, tiller number, and height were recorded.

### Estimating Bundle Sheath Leakiness Using Carbon Isotope Discrimination Coupled With Leaf Gas Exchange.

A LI-6800 gas exchange system (LI-COR Environmental) with the large light source chamber (6800-03) was coupled to a tunable-diode laser absorption spectroscope (TDLAS model TGA 200A; Campbell Scientific) to measure online carbon isotope discrimination as described previously (24). The TDL was connected to the LI-6800 reference air stream using the port on the back of the sensor head, and connected to the sample air stream using the port on the front of the sensor head. CO_2_-free air (N_2_/O_2_) with 21% O_2_ was created by mixing two gas streams with mass flow controllers (OMEGA Engineering Inc.). This air supplied the gas exchange system and was used in the calibration to correct for any drift in the TDL during measurements. The TDL was calibrated using a 2-point calibration where a linear fit is applied using the measurements from two calibration tanks with known [^12^CO_2_], [^13^CO_2_] (NOAA Global Monitoring Laboratory). The measurements cycled through four gas streams in the following sequence: calibration points 1,500 and 100 ppm CO_2_ (NOAA Global Monitoring Laboratory), LI-COR 6800 reference air stream, leaf chamber air stream, and reference air stream a second time. Each step had a duration of 30 s, except for the leaf chamber air, which had a duration of 600 s for a total cycle time of 720 s. Measurements were averaged over the last 10 s to produce a single data point for the calibration and reference measurements each cycle. For the sample line, measurements were averaged over 10 s intervals, excluding the first 10 s, to have 59 data points each cycle.

The gas exchange system was set to the following conditions: light intensity of 0 µmol m^−2^ s^−1^, leaf temperature of 27 °C, flow rate of 300 µmol s^−1^, 21% O_2_, and 800 µmol mol^−1^ CO_2._ Once steady-state respiration was reached, five consecutive measurements were logged to measure dark respiration. Afterward, the system was initiated to autolog at 10 s intervals over the course of 30 min. Light intensity increased to 1,800 µmol m^−2^ s^−1^ upon initiation of the autoprogram. Sorghum leaves from the field were cut from plants predawn, submerged in water and brought to the lab where they were recut underwater and left in the dark until measured. Only steady-state measurements of leakiness were calculated (25 to 30 min after light turned on).

Observed photosynthetic discrimination (Δ^13^C) was derived and bundle sheath leakiness (*ϕ*) was subsequently calculated from gas exchange parameters and Δ^13^C as described previously (67, 68). Data processing and calculations of Δ^13^C and *ϕ* were done using the MATLAB tool developed previously (24), with minor modifications to apply the 2-point calibration discussed above.

### Carbon and Nitrogen Analysis.

Leaf tissue from field-grown sorghum was oven dried to a constant weight, ground, and total carbon (C), C isotope analysis (δ15C), total nitrogen (N), and N isotope analysis (δ15 N) measured using an elemental analyzer (Costech Elemental Combustion System 4010, Costech Analytical Technologies) coupled with a continuous flow isotope ratio mass spectrometer (DeltaV Advantage, Thermo Fisher Scientific). Spinach leaves (National Institute of Standards and Technology Standard Reference Material 1570a) and acetanilide (USA Analytical) were used as standards for C and N analysis. Isotope analysis was measured relative to the international standards NBS 19 for C (δ^13^C_V-PDB_ = 1.95*‰)* and atmospheric N_2_ for N *(*δ^15^N_Air_ = 0‰).

### Statistical Analysis.

Normality of the data was tested with Shapiro–Wilk’s test, and homoscedasticity with Brown–Forsythe’s test. If criteria for normal distributions and equal variance was met one-way ANOVA followed by Dunnett’s post hoc test for transgenic mean comparison against the WT control was performed. Data were considered significant at *P* < 0.05, and at *P* < 0.1 when specified. If criteria for normality was violated Wilcoxon’s nonparametric test was applied. Analysis of field growth traits (Fig. 4 and *SI Appendix*, Table S2) was performed using a randomized block design with 6 blocks. Statistical tests used are indicated in each figure or table legend. Jmp pro version 17.0.0 software was used for all statistical analyses.

## Supplementary Material

Appendix 01 (PDF)

## Data Availability

Spreadsheets (.CSV, .xlsx) and R scripts (.R) data have been deposited in https://github.com/cabbi-bio/sorghum-sugarcane-RBCS-RAF1-2024 Github (69). All other data are included in the article and/or *SI Appendix*.

## References

[1] FAO, FAOSTAT (Food and Agricultural Organization of the United Nations, Rome, Italy, 2024).

[2] A. L. Healey , The complex polyploid genome architecture of sugarcane. Nature, **628**, 804–810 (2024), 10.1038/s41586-024-07231-4. 38538783 PMC11041754

[3] F. G. Dohleman, S. P. Long, More productive than maize in the midwest: How does miscanthus do it? Plant Physiol. **150**, 2104–2115 (2009). 19535474 10.1104/pp.109.139162 PMC2719137

[4] Y. He, D. Jaiswal, S. P. Long, X.-Z. Liang, M. L. Matthews, Biomass yield potential on U.S. marginal land and its contribution to reach net-zero emission. GCB Bioenergy **16**, e13128 (2024).

[5] E. A. Heaton, F. G. Dohleman, S. P. Long, Meeting US biofuel goals with less land: The potential of Miscanthus. Global Change Biol. **14**, 2000–2014 (2008).

[6] S. Matsuoka, A. J. Kennedy, E. G. D. d. Santos, A. L. Tomazela, L. C. S. Rubio, Energy cane: Its concept, development, characteristics, and prospects. Adv. Bot., **2014**, 597275 (2014).

[7] S. von Caemmerer, Updating the steady-state model of C4 photosynthesis. J. Exp. Bot. **72**, 6003–6017 (2021). 34173821 10.1093/jxb/erab266 PMC8411607

[8] B. J. Walker, A. VanLoocke, C. J. Bernacchi, D. R. Ort, The costs of photorespiration to food production now and in the future. Annu. Rev. Plant Biol. **67**, 107–127 (2016). 26865340 10.1146/annurev-arplant-043015-111709

[9] M. Ermakova , Installation of C4 photosynthetic pathway enzymes in rice using a single construct. Plant Biotechnol. J. **19**, 575–588 (2021). 33016576 10.1111/pbi.13487 PMC7955876

[10] R. Furbank, S. Kelly, S. von Caemmerer, Photosynthesis and food security: The evolving story of C4 rice. Photosynthesis Res. **158**, 121–130 (2023). 10.1007/s11120-023-01014-0 PMC10108777 37067631

[11] M. E. Bianconi , Continued adaptation of C4 photosynthesis after an initial burst of changes in the Andropogoneae grasses. Systematic Biol. **69**, 445–461 (2019). 10.1093/sysbio/syz066 PMC7672695 31589325

[12] E. A. Ainsworth, S. P. Long, What have we learned from 15 years of free-air CO2 enrichment (FACE)? A meta-analytic review of the responses of photosynthesis, canopy. New Phytologist **165**, 351–371 (2005). 15720649 10.1111/j.1469-8137.2004.01224.x

[13] E. A. Ainsworth, S. P. Long, 30 years of free-air carbon dioxide enrichment (FACE): What have we learned about future crop productivity and its potential for adaptation? Global Change Biol. **26**, 27–49 (2020). 10.1111/gcb.15375 33135850

[14] A. D. B. Leakey, C. J. Bernacchi, F. G. Dohleman, D. R. Ort, S. P. Long, Will photosynthesis of maize (Zea mays) in the US Corn Belt increase in future CO2 rich atmospheres? An analysis of diurnal courses of CO2 uptake under free-air concentration enrichment (FACE). Global Change Biol. **10**, 951–962 (2004).

[15] A. D. B. Leakey , Photosynthesis, productivity, and yield of maize are not affected by open-air elevation of CO2 concentration in the absence of drought. Plant Physiol. **140**, 779–790 (2006). 16407441 10.1104/pp.105.073957 PMC1361343

[16] A. P. de Souza, R. A. Arundale, F. G. Dohleman, S. P. Long, M. S. Buckeridge, Will the exceptional productivity of Miscanthus x giganteus increase further under rising atmospheric CO2? Agri. Forest Meteorol. **171**, 82–92 (2013).

[17] R. F. Sage, D. S. Kubien, Quo vadis C4? An ecophysiological perspective on global change and the future of C4 plants. Photosynth Res. **77**, 209–225 (2003). 16228377 10.1023/A:1025882003661

[18] R. T. Furbank, Walking the C4 pathway: Past, present, and future. J. Exp. Bot. **67**, 4057–4066 (2016). 27059273 10.1093/jxb/erw161

[19] C. P. Pignon, S. P. Long, Retrospective analysis of biochemical limitations to photosynthesis in 49 species: C4 crops appear still adapted to pre-industrial atmospheric [CO2]. Plant Cell Environ. **43**, 2606–2622 (2020). 32743797 10.1111/pce.13863

[20] S. von Caemmerer, R. T. Furbank, Strategies for improving C4 photosynthesis. Curr. Opin. Plant Biol. **31**, 125–134 (2016). 27127850 10.1016/j.pbi.2016.04.003

[21] NOAA, Trends in Atmospheric Carbon Dioxide. (Global Monitoring Laboratory, NOAA, U.S. Department of Commerce, Boulder CO, 2024).

[22] J. Ahn , A record of atmospheric CO2 during the last 40,000 years from the Siple Dome, Antarctica ice core. J. Geophys. Res.: Atmos. **109**, D13305 (2004).

[23] Y. Wang, K. X. Chan, S. P. Long, Towards a dynamic photosynthesis model to guide yield improvement in C4 crops. Plant J. **107**, 343–359 (2021). 34087011 10.1111/tpj.15365 PMC9291162

[24] Y. Wang , Increased bundle-sheath leakiness of CO2 during photosynthetic induction shows a lack of coordination between the C4 and C3 cycles. New Phytologist **236**, 1661–1675 (2022). 36098668 10.1111/nph.18485 PMC9827928

[25] R. T. Furbank , Genetic manipulation of key photosynthetic enzymes in the C4 plant Flaveria bidentis. Funct. Plant Biol. **24**, 477–485 (1997).

[26] C. E. Salesse-Smith , Overexpression of Rubisco subunits with RAF1 increases Rubisco content in maize. Nat. Plant. **4**, 802–810 (2018). 10.1038/s41477-018-0252-4 30287949

[27] C. E. Salesse-Smith, R. E. Sharwood, F. A. Busch, D. B. Stern, Increased Rubisco content in maize mitigates chilling stress and speeds recovery. Plant Biotechnol. J. **18**, 1409–1420 (2020). 31793172 10.1111/pbi.13306 PMC7207003

[28] D. K. Yoon , Transgenic rice overproducing Rubisco exhibits increased yields with improved nitrogen-use efficiency in an experimental paddy field. Nat. Food **1**, 134–139 (2020). 37127998 10.1038/s43016-020-0033-x

[29] S. Von Caemmerer, Biochemical models of leaf photosynthesis (Csiro Publishing, 2000).

[30] S. P. Long , Into the shadows and back into sunlight: Photosynthesis in fluctuating light. Annu. Rev. Plant Biol. **73**, 617–648 (2022). 35595290 10.1146/annurev-arplant-070221-024745

[31] R. A. Slattery, B. J. Walker, A. P. M. Weber, D. R. Ort, The impacts of fluctuating light on crop performance. Plant Physiol. **176**, 990–1003 (2018). 29192028 10.1104/pp.17.01234 PMC5813574

[32] U. Feller, I. Anders, T. Mae, Rubiscolytics: Fate of Rubisco after its enzymatic function in a cell is terminated. J. Exp. Bot. **59**, 1615–1624 (2007). 17975207 10.1093/jxb/erm242

[33] A. I. Flamholz , Revisiting Trade-offs between Rubisco kinetic parameters. Biochemistry **58**, 3365–3376 (2019). 31259528 10.1021/acs.biochem.9b00237 PMC6686151

[34] R. F. Sage, A. D. McKown, Is C4 photosynthesis less phenotypically plastic than C3 photosynthesis? J. Exp. Bot. **57**, 303–317 (2005). 16364950 10.1093/jxb/erj040

[35] C. P. Pignon, M. R. Lundgren, C. P. Osborne, S. P. Long, Bundle sheath chloroplast volume can house sufficient Rubisco to avoid limiting C4 photosynthesis during chilling. J. Exp. Bot. **70**, 357–365 (2018). 10.1093/jxb/ery345 PMC6305190 30407578

[36] G. Zhang , The reference genome of Miscanthus floridulus illuminates the evolution of Saccharinae. Nat. Plant. **7**, 608–618 (2021). 10.1038/s41477-021-00908-y PMC8238680 33958777

[37] R. F. Sage, Was low atmospheric CO_2_ during the Pleistocene a limiting factor for the origin of agriculture? Global Change Biol. **1**, 93–106 (1995).

[38] K. Wostrikoff, D. Stern, Rubisco large-subunit translation is autoregulated in response to its assembly state in tobacco chloroplasts. Proc. Natl. Acad. Sci. U.S.A. **104**, 6466–6471 (2007). 17404229 10.1073/pnas.0610586104 PMC1851044

[39] Y. Suzuki, A. Makino, Availability of rubisco small subunit up-regulates the transcript levels of large subunit for stoichiometric assembly of its holoenzyme in rice. Plant Physiol. **160**, 533–540 (2012). 22811433 10.1104/pp.112.201459 PMC3440226

[40] Y. Suzuki , Increased Rubisco content in transgenic rice transformed with the ‘Sense’ rbcS Gene. Plant Cell Physiol. **48**, 626–637 (2007). 17379698 10.1093/pcp/pcm035

[41] C. Hermida-Carrera, M. V. Kapralov, J. Galmés, Rubisco catalytic properties and temperature response in crops. Plant Physiol. **171**, 2549–2561 (2016). 27329223 10.1104/pp.16.01846 PMC4972260

[42] C. Ishikawa, T. Hatanaka, S. Misoo, C. Miyake, H. Fukayama, Functional incorporation of sorghum small subunit increases the catalytic turnover rate of rubisco in transgenic rice. Plant Physiol. **156**, 1603–1611 (2011). 21562335 10.1104/pp.111.177030 PMC3135941

[43] N. Salvatori, G. Alberti, O. Muller, A. Peressotti, Does fluctuating light affect crop yield? A focus on the dynamic photosynthesis of two soybean varieties. Front. Plant Sci. **13**, 862275 (2022). 35557734 10.3389/fpls.2022.862275 PMC9085482

[44] J. Kromdijk , Improving photosynthesis and crop productivity by accelerating recovery from photoprotection. Science **354**, 857–861 (2016). 27856901 10.1126/science.aai8878

[45] S. H. Taylor , Faster than expected Rubisco deactivation in shade reduces cowpea photosynthetic potential in variable light conditions. Nat. Plant. **8**, 118–124 (2022). 10.1038/s41477-021-01068-9 PMC8863576 35058608

[46] S. H. Taylor, S. P. Long, Slow induction of photosynthesis on shade to sun transitions in wheat may cost at least 21% of productivity. Philos. Trans. R Soc. Lond. B Biol. Sci. **372**, 1730 (2017). 10.1098/rstb.2016.0543 PMC5566890 28808109

[47] S. Deschamps , A chromosome-scale assembly of the sorghum genome using nanopore sequencing and optical mapping. Nat. Commun. **9**, 4844 (2018). 30451840 10.1038/s41467-018-07271-1 PMC6242865

[48] S. Belide, T. Vanhercke, J. R. Petrie, S. P. Singh, Robust genetic transformation of sorghum (Sorghum bicolor L.) using differentiating embryogenic callus induced from immature embryos. Plant Methods **13**, 109 (2017). 29234458 10.1186/s13007-017-0260-9 PMC5723044

[49] F. R. Miller, Registration of RTX430 sorghum parental line. Crop Science **24**, 1224–1224 (1984).

[50] A. B. Cousins, Development of C4 photosynthesis in sorghum leaves grown under free-air CO2 enrichment (FACE). J. Exp. Bot. **54**, 1969–1975 (2003). 12837815 10.1093/jxb/erg197

[51] A. B. Cousins , Reduced photorespiration and increased energy-use efficiency in young CO2-enriched sorghum leaves. New Phytologist **150**, 275–284 (2001).

[52] G. W. Wall , Elevated atmospheric CO2 improved *Sorghum* plant water status by ameliorating the adverse effects of drought. New Phytologist **152**, 231–248 (2001).

[53] M. J. Ottman , Elevated CO2 increases sorghum biomass under drought conditions. New Phytologist **150**, 261–273 (2001).

[54] J. Sheen, Metabolic repression of transcription in higher plants. Plant Cell **2**, 1027–1038 (1990). 2136626 10.1105/tpc.2.10.1027 PMC159951

[55] X. M. Guo, Z. X. Ge, S. J. Sato T. E. Clemente, “Sorghum: Sorghum bicolor” in Agrobacterium Protocols, **Vol 1**, 3rd Edition, K. Wang, Ed. (2015), vol. **1223**, pp. 181–188. 10.1007/978-1-4939-1695-5_14 25300840

[56] K. Głowacka , An evaluation of new and established methods to determine T-DNA copy number and homozygosity in transgenic plants. Plant Cell Amp. Environ. **39**, 908–917 (2016). 10.1111/pce.12693 PMC5021166 26670088

[57] S. Belide, T. Vanhercke, J. R. Petrie, S. P. Singh, Robust genetic transformation of sorghum (Sorghum bicolor L.) using differentiating embryogenic callus induced from immature embryos. Plant Methods **13** (2017). 10.1186/s13007-017-0260-9 PMC5723044 29234458

[58] Y. Taparia, M. Gallo, F. Altpeter, Comparison of direct and indirect embryogenesis protocols, biolistic gene transfer and selection parameters for efficient genetic transformation of sugarcane. Plant Cell Tissue Organ Culture (PCTOC) **111**, 131–141 (2012).

[59] F. Altpeter, S. Sandhu, “Genetic transformation–biolistics” in Plant Cell Culture: Essential Methods, M. Davey, P. Anthony, Eds. (Wiley, Chichester, 2010), pp. 217–239. 10.1002/9780470686522.ch12.

[60] M. G. Murray, W. F. Thompson, Rapid isolation of high molecular weight plant DNA. Nucleic Acids Res. **8**, 4321–4325 (1980). 7433111 10.1093/nar/8.19.4321 PMC324241

[61] C. E. Salesse-Smith , Greater mesophyll conductance and leaf photosynthesis in the field through modified cell wall porosity and thickness via AtCGR3 expression in tobacco. Plant Biotechnol. J., **22**, 2504–2517 (2024), 10.1111/pbi.14364. 38687118 PMC11331791

[62] S. Ruuska , The interplay between limiting processes in C-3 photosynthesis studied by rapid-response gas exchange using transgenic tobacco impaired in photosynthesis. Aust. J. Plant Physiol. **25**, 859–870 (1998).

[63] R. E. Sharwood, S. Von Caemmerer, P. Maliga, S. M. Whitney, The catalytic properties of hybrid rubisco comprising tobacco small and sunflower large subunits mirror the kinetically equivalent source rubiscos and can support tobacco growth. Plant Physiol. **146**, 83–96 (2008). 17993544 10.1104/pp.107.109058 PMC2230571

[64] S. M. Whitney, R. E. Sharwood, “Rubisco engineering by plastid transformation and protocols for assessing expression” in Chloroplast Biotechnology: Methods and Protocols, P. Maliga, Ed. (Springer US, New York, NY, 2021), pp. 195–214. 10.1007/978-1-0716-1472-3_10. 34028770

[65] M. A. J. Parry , Regulation of Rubisco by inhibitors in the light. Plant Cell Amp. Environ. **20**, 528–534 (1997).

[66] E. B. Lochocki, PhotoGEA: Photosynthetic Gas Exchange Analysis. v.0.11.0 (2024).

[67] J. R. Evans, T. D. Sharkey, J. A. Berry, G. D. Farquhar, Carbon isotope discrimination measured concurrently with gas-exchange to investigate CO2 diffusion in leaves of higher-plants. Aus. J. Plant Physiol. **13**, 281–292 (1986).

[68] S. von Caemmerer, O. Ghannoum, J. J. L. Pengelly, A. B. Cousins, Carbon isotope discrimination as a tool to explore C4 photosynthesis. J. Exp. Bot. **65**, 3459–3470 (2014). 24711615 10.1093/jxb/eru127

[69] C. E. Salesse-Smith , Sorghum-sugarcane-RBCS-RAF1-2024. GitHub. https://github.com/cabbi-bio/sorghum-sugarcane-RBCS-RAF1-2024. Deposited 21 January 2025.

