# Supplementary material for: Adapting C4 photosynthesis to atmospheric change and increasing productivity by elevating Rubisco content in sorghum and sugarcane
Source: Proc Natl Acad Sci U S A. 2025 Feb 11;122(8):e2419943122. doi: 10.1073/pnas.2419943122 (PMC11873827; doi:10.1073/pnas.2419943122)
Supplement: Supplementary file 1 — Appendix 01 (PDF) [file pnas.2419943122.sapp.pdf]

SUPPLEMENTAL DATA

Adapting C_4 photosynthesis to atmospheric change and increasing productivity by elevating Rubisco content in Sorghum and Sugarcane.

Coralie E. Salesse-Smith[1,3], Noga Adar[1], Baskaran Kannan[4,5], Thaibinhduong Nguyen[4,5], Wei Wei[1], Ming Guo[6,7], Zhengxiang Ge[6,7], Fredy Altpeter[4,5], Tom E. Clemente[6,7], and Stephen P. Long[1,2,3]

[1]Carl R. Woese Institute for Genomic Biology, University of Illinois at Urbana-Champaign, Urbana, IL, USA; [2]Departments of Plant Biology and of Crop Sciences, University of Illinois at Urbana-Champaign, Urbana, IL, USA; [3]DOE Center for Advanced Bioenergy and Bioproducts Innovation, Urbana-Champaign, IL, USA; [4]Agronomy Department, Plant Molecular and Cellular Biology Program, Genetics Institute, University of Florida, IFAS, Gainesville, Florida, USA; [5]DOE Center for Advanced Bioenergy and Bioproducts Innovation, Gainesville, FL, USA; [6]Department of Agronomy and Horticulture, University of Nebraska-Lincoln, Lincoln, NE, USA; [7]DOE Center for Advanced Bioenergy and Bioproducts Innovation, Lincoln, NE, USA

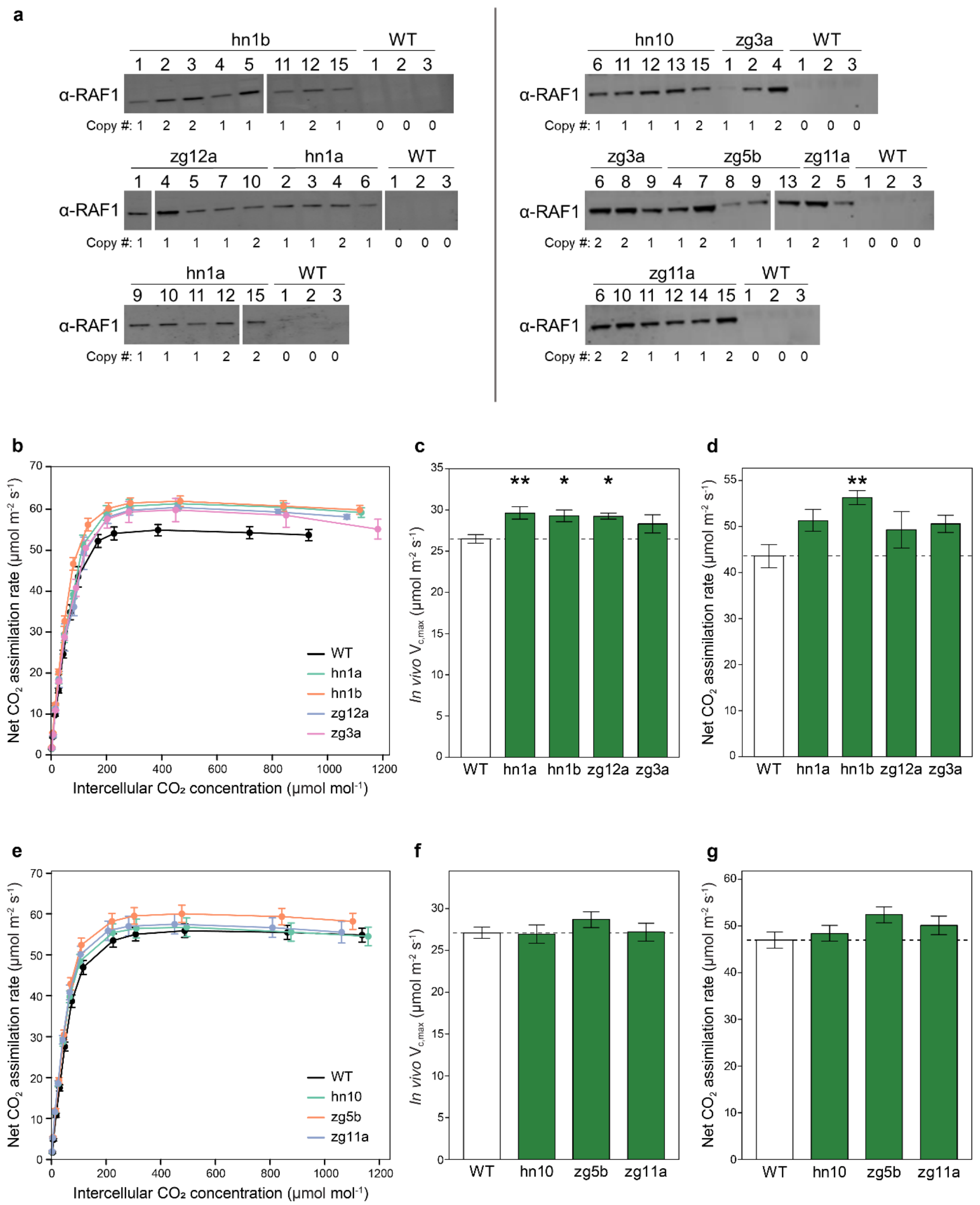

Fig. S1: Screening T1 transgenic sorghum lines.

(a) Total soluble protein isolated on a leaf area basis from sorghum, analyzed by immunoblot and probed with anti-RAF1 antibody. Copy number of NPTII obtained by digital droplet PCR is indicated below each sample. **(b and e)** Response of net CO_2 assimilation (A_{sat}) to intercellular [CO2]. **(c and f)** Maximum *in vivo* Rubisco carboxylation rate ($V_{c,max}$) at 25 °C estimated from response curves. **(d and g)** A_{sat} measured at 400 µmol mol^{-1} CO_2. Values are shown as the mean ± SEM. Asterisks indicate significant differences between WT and the transgenic line (**P < 0.05, *P<0.1); one-way ANOVA, Dunnett's post hoc test. For gas exchange screening the seven transgenic events were split into two sets. Set 1 (b-d) n=4. Set 2 (e-f) n=7-8.

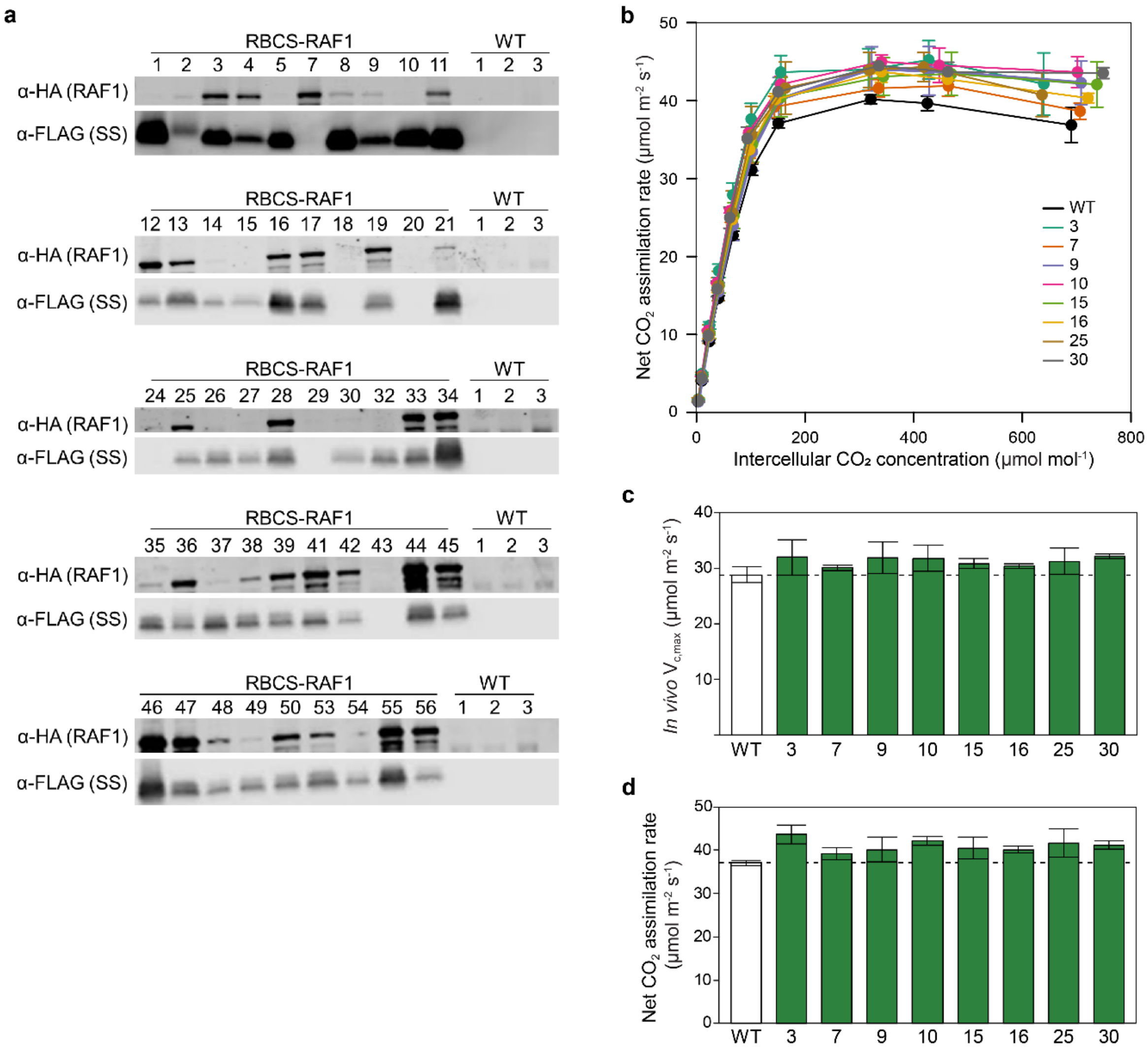

Fig. S2: Screening T0 transgenic sugarcane lines.

(a) Total soluble protein isolated on a leaf area basis from sugarcane, analyzed by immunoblot and probed with antibodies indicated to the left. **(b)** Response of net CO_2 assimilation (A_{sat}) to intercellular [CO2]. **(c)** Maximum *in vivo* Rubisco carboxylation rate ($V_{c,max}$) at 25 °C estimated from response curves (d) A_{sat} measured at 400 µmol mol^{-1} CO_2. Values are shown as the mean ± SEM (n=3 technical replicates from different leaves).

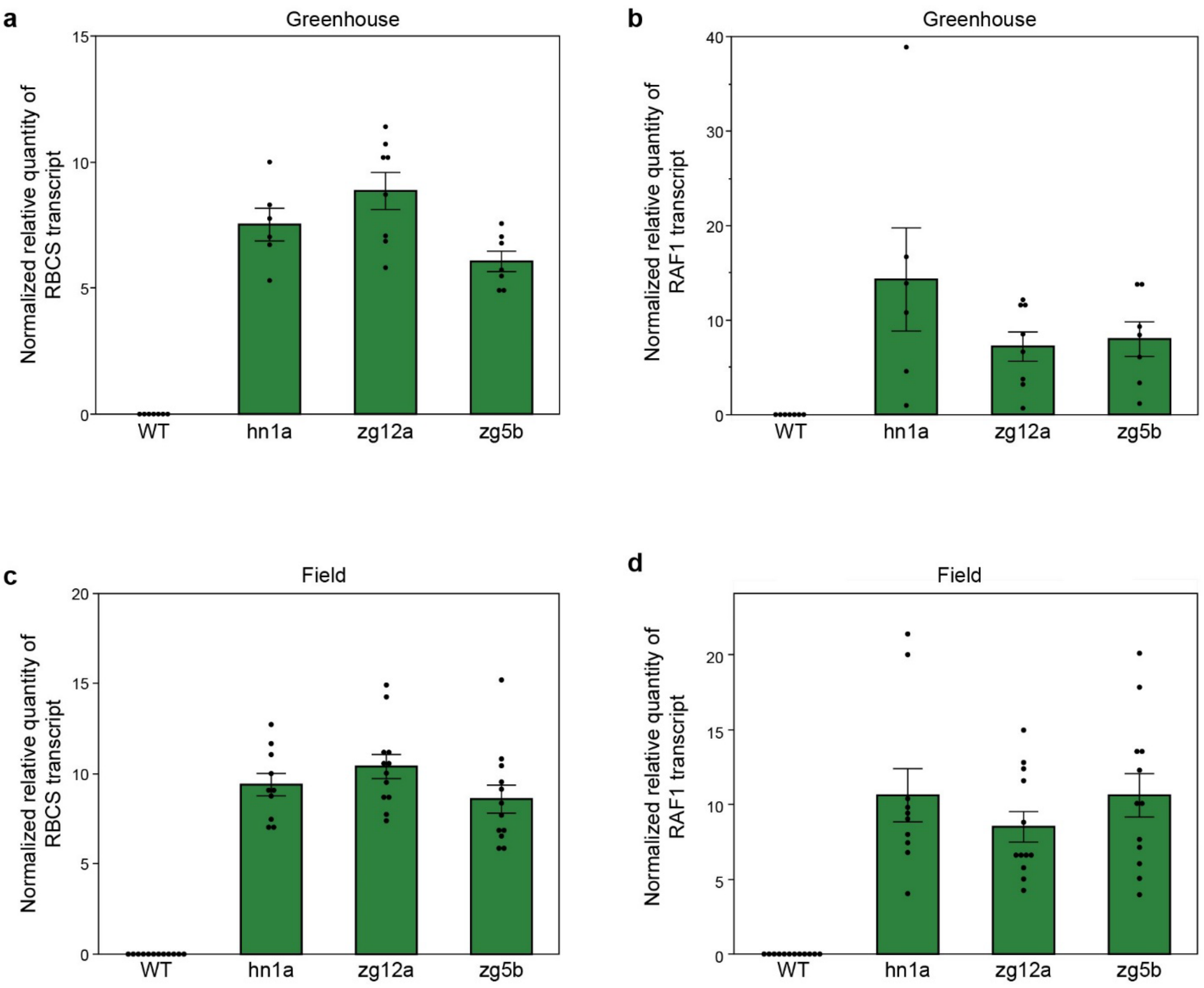

Fig. S3: Gene expression in greenhouse and field grown sorghum.

(a) qPCR analysis of *ZmRbcS* and **(b)** *ZmRaf1* gene expression in three independent transgenic sorghum events grown in the greenhouse (n = 6-8). **(c)** *ZmRbcS* and **(d)** *ZmRaf1* gene expression in field grown sorghum (n = 10-12). Values are shown as the mean $\pm$ SEM.

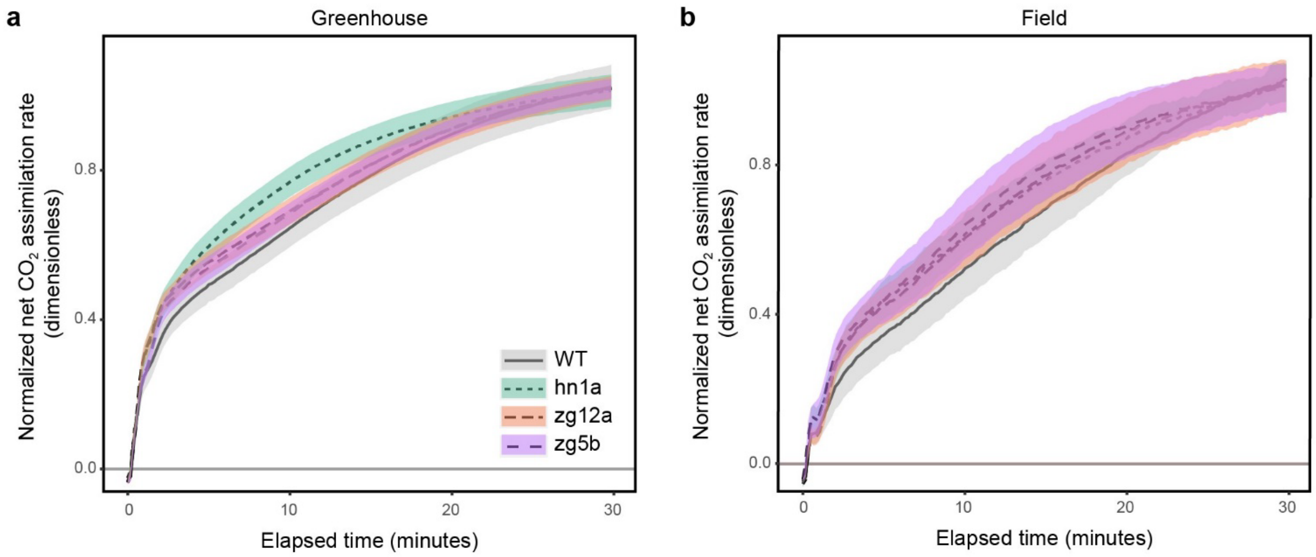

Fig. S4: Normalized induction curves from greenhouse and field grown sorghum.

(a) Normalized induction of net CO_2 assimilation during the first 30 min of illumination at PPFD 1800 µmol m^{-2} s^{-1} in the greenhouse (n = 8-9) and **(b)** in field grown sorghum (n = 4-5). Values are shown as the mean ± SEM and were normalized by the average value over the last 5 minutes of illumination from Fig. 2e and 3b respectively.

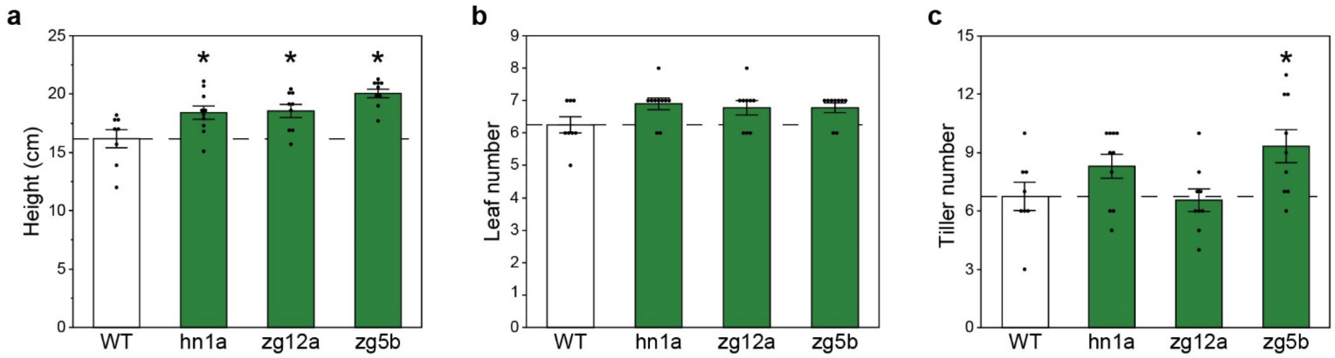

Fig. S5: Plant growth traits in greenhouse grown sorghum plants.

(a) Plant height, **(b)** leaf number, **(c)** tiller number. Values are shown as the mean ± SEM (n = 8-10). Asterisks indicate significant differences between WT and the transgenic line (*P < 0.05); one-way ANOVA, Dunnett's post hoc test.

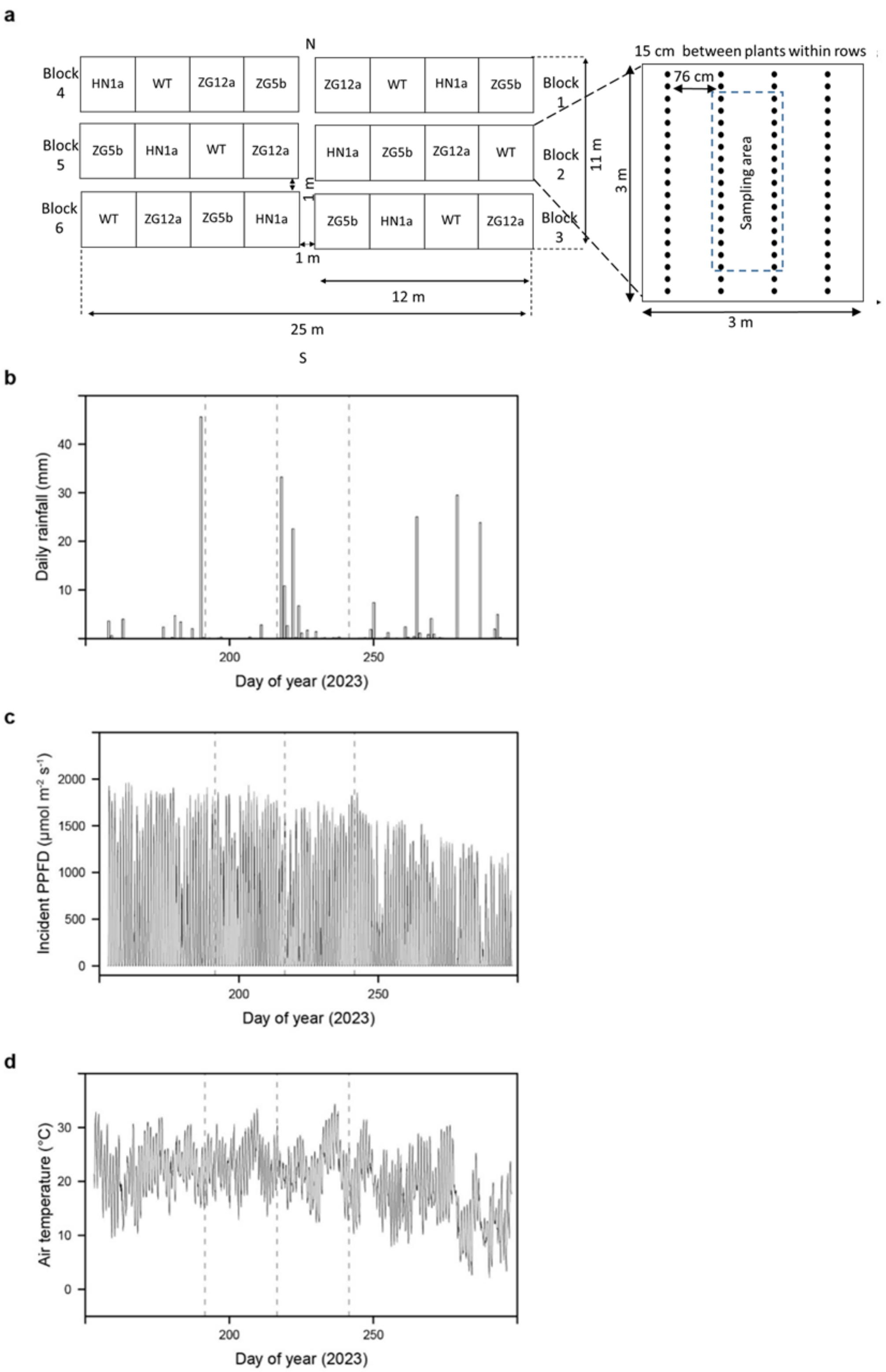

Fig. S6: Sorghum field experimental design and weather conditions.

(a) Schematic of field experimental set up. A randomized block design was used. Genotypes were randomly assigned to one of four positions in each of the six blocks; ● on the inset shows the location of individual plants. **(b)** Total daily rainfall, **(c)** light intensity and **(d)** air temperature from June 2nd (DOY 153 - date sorghum sowed) until October 4th, 2023 (DOY 297 - date sorghum harvested). (c-d) Data are shown as averages over the preceding hour. Dashed vertical lines show dates of gas exchange measurements; July 10th (DOY 191), August 4th (DOY 216), and August 29th (DOY 241).

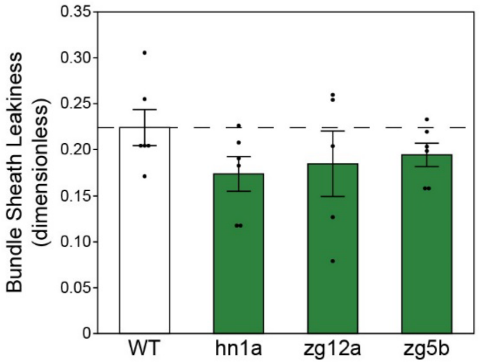

Fig. S7: Steady-state BS leakiness of field grown sorghum.

Bundle-sheath leakiness measurements. Values are shown as the mean ± SEM (n = 5-6). No significant differences, one-way ANOVA, Dunnett's post hoc test.

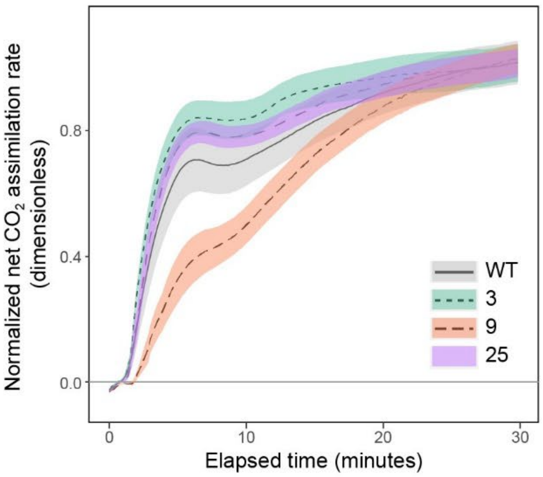

Fig. S8: Normalized induction curves from greenhouse-grown sugarcane.

(a) Normalized induction of net CO_2 assimilation during the first 30 min of illumination at PPFD 1800 µmol m^{-2} s^{-1} (n = 7-8). Values are shown as the mean ± SEM and were normalized by the average value over the last 5 minutes of illumination from Fig. 5b.

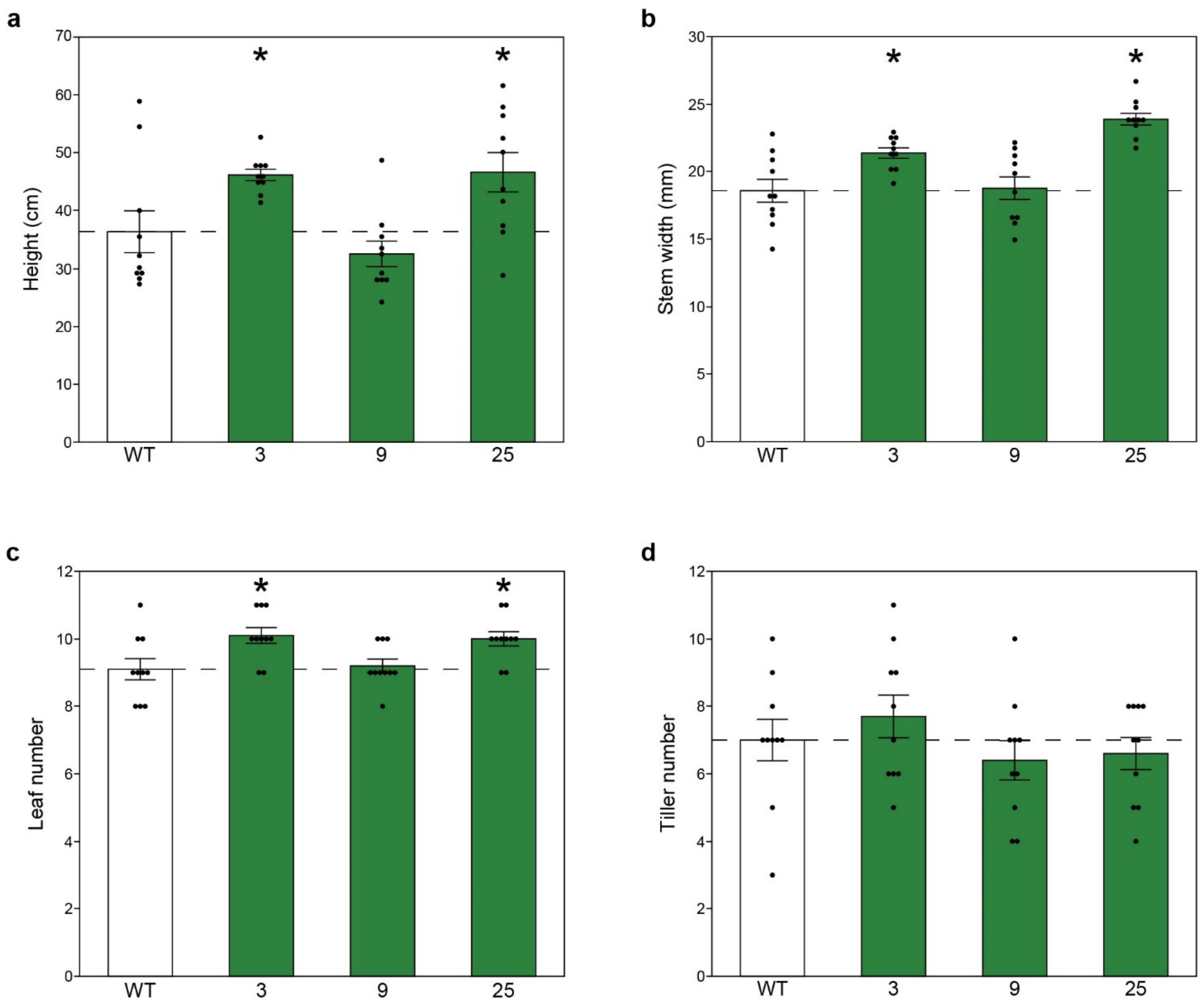

Fig. S9: Plant growth traits in greenhouse grown sugarcane plants.

(a) Plant height, **(b)** stem width, **(c)** leaf number and **(d)** tiller number. Values are shown as the mean $\pm$ SEM (n = 10). Asterisks indicate significant differences between WT and the transgenic line (*P < 0.05); one-way ANOVA, Dunnett's post hoc test.

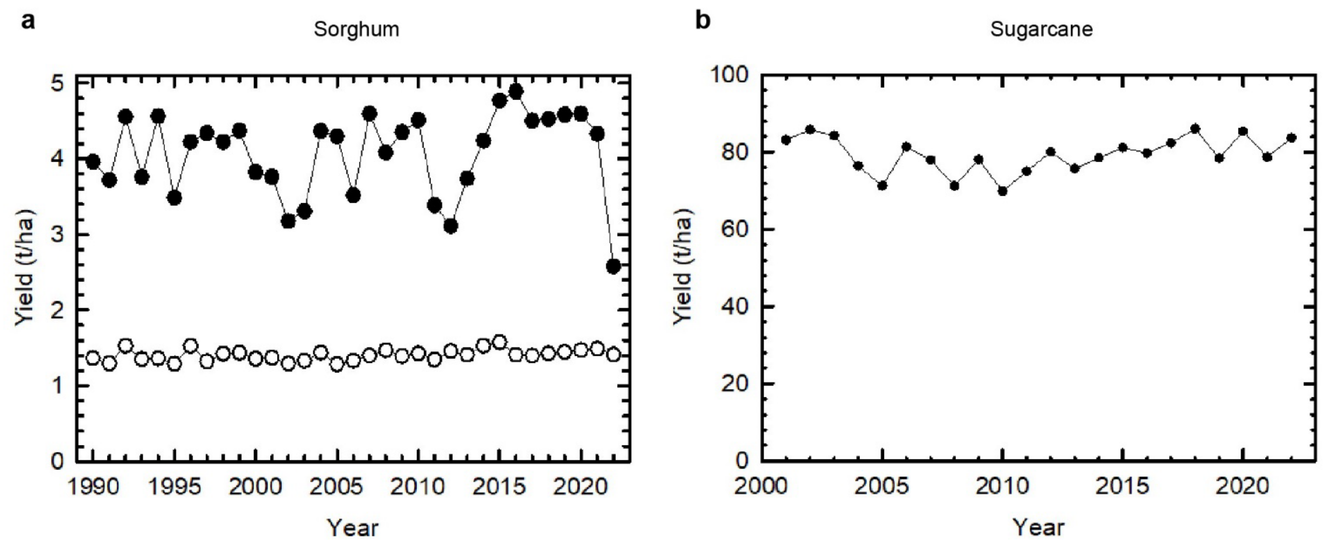

Fig. S10: Average yield of sorghum and sugarcane over the last 20 to 30 years.

(a) Official figures from FAOstat (2024) for average World (open symbols) and USA (closed symbols) sorghum grain yields for 1990-2022, and **(b)** yield of harvested sugarcane stalks in the USA from 2001-2022.

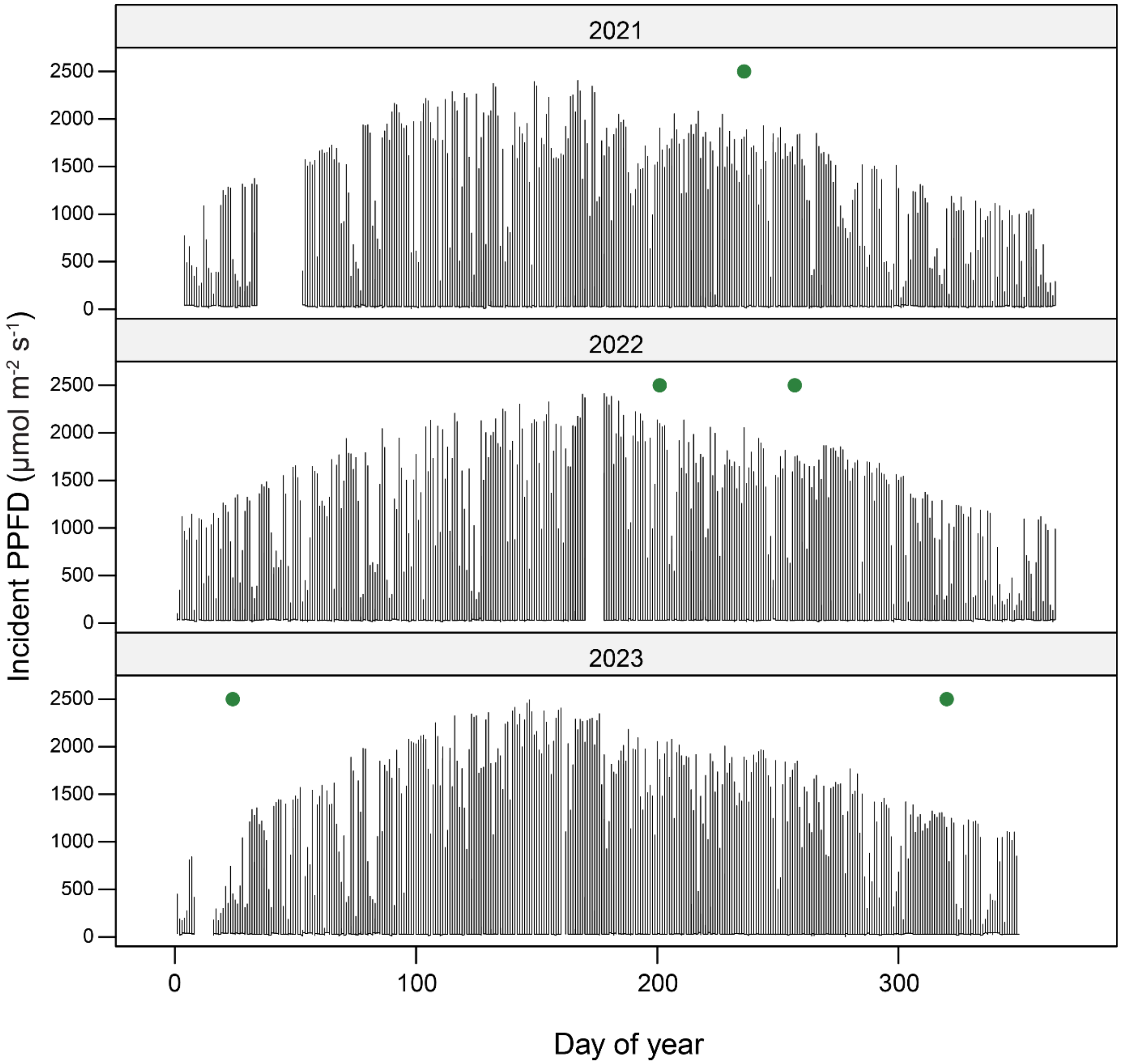

Fig. S11: Light intensity directly outside the greenhouse.

Outside irradiance data for greenhouse experiments presented in this manuscript. Filled green circles show dates for gas exchange measurements: sugarcane screening on August 24-27, 2021 (DOY 236-239); sorghum screening (set I) on July 20, 2022 (DOY 201); sorghum screening (set II) on September 14-15, 2022 (DOY 257-258); main sorghum experiment on January 24-26, 2023 (DOY 24-26); main sugarcane experiment on November 16-17, 2023 (DOY 320-321).

Table S1. List of primers used in this manuscript. * designates reference genes.

Primer name	Sequence	Type	Amplicon length (bp)
ddNPTII_F	TACGCTTGATCCGGCTAC	Sb ddPCR	77
ddNPTII_R	CTTCCATCCGAGTACGTG	Sb ddPCR	
*Sb ENOL-2_F	TGAGGACCCTTTTGATCAGG	Sb ddPCR	135
*Sb ENOL-2_R	CAAGCCTTCTTGCCAATAGC	Sb ddPCR	
So Construct_F	TAATGGTGGTGTAGCTCGACG	So PCR	209
So Construct_R	TGGCTTGGTTAGGTTTGGCT	So PCR	
*NPTII_F	TACCTGCCCATTCGACCACC	So PCR	345
*NPTII_R	TAAAGCACGAGGAAGCGGTC	So PCR	
ZmRBCS_F	GCGTCTCGAACTTCTTGTTGC	Sb qPCR	111
ZmRBCS_R	TGGACTGATGTGTGTTGCCC	Sb qPCR	
ZmRAF1_F	CCAAGGAGTCGAAGTAGGGAA	Sb qPCR	149
ZmRAF1_R	CTTCAACCCGTCTTCCATCCA	Sb qPCR	
*SbEIF4α_F	CAACTTTGTCACCCGCGATGA	Sb qPCR	144
*SbEIF4α_R	TCCAGAAACCTTAGCAGCCCA	Sb qPCR	
*SbActin11_F	AACTGCAGATGTGGATTGCCAAGG	Sb qPCR	115
*SbActin11_R	ATAATGGCTCCTCTCGGCTTGCAT	Sb qPCR	

Table S2. Summary of harvest measurements and yield characterization of field-grown sorghum. Values are shown as the mean ± SEM. Values in bold are significantly different from WT at $P < 0.05$.

Parameter	WT	RBCS-RAF1 Hn1a	RBCS-RAF1 Zg12a	RBCS-RAF1 Zg5b	Sample number (*N*)
**Sorghum - Field**					
Panicle dry weight, with seed (kg)	3.53 ± 0.15	2.99 ± 0.23	3.24 ± 0.12	3.24 ± 0.25	6 plots
Panicle dry weight, without seed (kg)	0.654 ± 0.030	0.657 ± 0.046	0.631 ± 0.023	0.648 ± 0.050	6 plots
Above ground biomass (kg)	8.10 ± 0.32	8.24 ± 0.53	8.34 ± 0.30	8.75 ± 0.66	6 plots
Number of panicles per plot	69.5 ± 2.36	70.7 ± 4.14	56.7 ± 3.28	60.8 ± 4.21	6 plots
100 seed weight (g)	4.02 ± 0.050	**3.78 ± 0.029**	**3.83 ± 0.033**	4.06 ± 0.018	6 plots
Average seed weight per panicle (g)	41.3 ± 1.03	**32.8 ± 1.19**	46.6 ± 2.81	42.5 ± 1.09	6 plots
Panicle emergence (DOY)	227 ± 0.48	**233 ± 0.34**	**230 ± 0.40**	**232 ± 0.54**	6 plots

Table S3. Summary of leaf mass per area (LMA), chlorophyll content (SPAD value), leaf carbon and nitrogen content and total soluble protein (TSP) of sorghum and sugarcane plants. (a) Relative abundance of LS protein estimated from Fig. 1b (sorghum) and Fig. 1c (sugarcane). Values are shown as the mean ± SEM. Values in bold are significantly different from WT at *P* < 0.05.

Parameter	WT	RBCS-RAF1 Hn1a	RBCS-RAF1 Zg12a	RBCS-RAF1 Zg5b	Sample number (*N*)
**Sorghum - Greenhouse**					
TSP (µg/ml)	289.02 ± 27.96	280.22 ± 25.68	300.22 ± 30.36	286.58 ± 25.09	8
Relative abundance[(a)] of LS protein	1 ± 0.35	1.49 ± 0.17	1.35 ± 0.30	1.30 ± 0.23	3
**Sorghum - Field**					
LMA (g/m^2)	55.18 ± 1.18	56.21 ± 0.84	54.80 ± 0.94	55.33 ± 0.80	12
SPAD	55.30 ± 2.47	51.52 ± 2.28	56.57 ± 2.35	52.28 ± 0.756	15
Leaf Carbon (%)	43.17 ± 0.32	42.91 ± 0.51	42.16 ± 0.61	42.67 ± 0.45	11-12
$\delta^{13}C$ (‰)	-12.17 ± 0.018	-12.23 ± 0.043	-12.18 ± 0.030	**-12.26 ± 0.021**	10-12
Leaf Nitrogen (%)	3.14 ± 0.081	3.21 ± 0.085	3.21 ± 0.060	3.18 ± 0.058	12
$\delta^{15}N$ (‰)	4.76 ± 0.21	4.68 ± 0.094	4.24 ± 0.14	4.92 ± 0.18	11-12

Parameter	WT	RBCS-RAF1 3	RBCS-RAF1 9	RBCS-RAF1 25	Sample number (*N*)
**Sugarcane - Greenhouse**					
LMA (g/m^2)	47.53 ± 2.99	42.19 ± 0.85	45.31 ± 1.32	41.50 ± 1.11	4-5
SPAD	55.06 ± 1.22	55.77 ± 0.83	58.00 ± 1.11	54.49 ± 1.10	10
TSP (µg/ml)	244.67 ± 9.28	245.67 ± 4.74	244.67 ± 8.65	246.67 ± 5.88	6
Relative abundance[(a)] of LS protein	1 ± 0.079	**1.20 ± 0.055**	0.91 ± 0.079	1.10 ± 0.038	3
